# SLC35D1 Is Associated with Colorectal Cancer Progression, EMT-Related Phenotypes, and Immune Infiltration

**DOI:** 10.3390/ijms27198517

**Published:** 2026-09-24

**Authors:** Yu-Jia Chang, Kuei-Yen Tsai, Crystal Ngofi Zumbi, Cheng-Chin Lee, G. M. Shazzad Hossain Prince, Uyanga Batzorig, Chien-Yu Huang, Po-Li Wei

**Affiliations:** 1 Graduate Institute of Clinical Medicine, College of Medicine, Taipei Medical University, Taipei 11031, Taiwan; r5424012@tmu.edu.tw (Y.-J.C.); leiftsai@tmu.edu.tw (K.-Y.T.); d339108005@tmu.edu.tw (C.N.Z.); d119110008@tmu.edu.tw (C.-C.L.); princegmsh@tmu.edu.tw (G.M.S.H.P.); 2 Center for Precision Medicine and Translational Cancer Research, Department of Medical Research, Taipei Medical University Hospital, Taipei Medical University, Taipei 11031, Taiwan; 3 Department of Pathology, Wan Fang Hospital, Taipei Medical University, Taipei 116079, Taiwan; 4 Cell Physiology and Molecular Image Research Center, Wan Fang Hospital, Taipei Medical University, Taipei 116079, Taiwan; 5 Department of Surgery, School of Medicine, College of Medicine, Taipei Medical University, Taipei 11031, Taiwan; 6 Division of General Surgery, Department of Surgery, Shuang Ho Hospital, Taipei Medical University, New Taipei City 235041, Taiwan; 7 Department of Dermatology, Department of Cellular and Molecular Medicine, UCSD Stem Cell Program, University of California, San Diego, La Jolla, CA 92093, USA; ubatzorig@health.ucsd.edu; 8 School of Medicine, National Tsing Hua University, Hsinchu 300044, Taiwan; 9 Institute of Molecular and Cellular Biology, National Tsing Hua University, Hsinchu 300044, Taiwan; 10 Division of Colorectal Surgery, Department of Surgery, Taipei Medical University Hospital, Taipei Medical University, Taipei 11031, Taiwan; 11 Graduate Institute of Cancer Biology and Drug Discovery, Taipei Medical University, Taipei 11031, Taiwan; 12 Taipei Cancer Center, Taipei Medical University, Taipei 11031, Taiwan

**Keywords:** colorectal cancer, nucleotide-sugar transporters, SLC35D gene family, SLC35D1, immune cell infiltration, epithelial–mesenchymal transition (EMT)

## Abstract

Colorectal cancer (CRC) is one of the most common malignancies worldwide, and the prognosis of patients with metastatic disease remains poor. Solute carrier proteins participate in diverse cellular processes, yet the biological and clinical relevance of the SLC35D family in CRC remains largely unexplored. This study systematically investigated the expression patterns and clinical significance of SLC35D family members and further evaluated the functional role of SLC35D1 in CRC. Bioinformatic analyses using TCGA and other publicly available datasets and analytical platforms were performed to evaluate the expression patterns and clinical significance of SLC35D family members in CRC. Associations of SLC35D1 expression with patient survival, immune cell infiltration, immune checkpoint markers, and cancer-related transcriptional programs were examined. Loss- and gain-of-function experiments in CRC cells were performed to assess the effects of SLC35D1 on cell proliferation, migration, invasion, and EMT-related phenotypes. SLC35D1 expression was significantly decreased in CRC tissues, SLC35D2 showed a nonsignificant downward trend, and SLC35D3 was significantly increased. Lower SLC35D1 expression was associated with poorer clinical outcomes in the TCGA cohort, although this prognostic association was not reproduced in the independent GSE39582 cohort. Bioinformatic analyses further revealed associations between SLC35D1 expression and immune cell infiltration, immune checkpoint markers, and several cancer-related transcriptional programs. Although the association between SLC35D1 and the EMT-related signature was modest, pathway-enrichment analyses suggested a potential relationship with EMT-related processes. Consistent with these observations, loss- and gain-of-function studies showed that SLC35D1 expression was associated with changes in CRC cell proliferation, migration, invasion, and EMT-related molecular phenotypes. Our findings identify SLC35D1 as an understudied SLC35D family member associated with CRC progression and clinical outcome. Functional studies showed that modulation of SLC35D1 expression was associated with changes in malignant phenotypes in CRC cells, whereas its relationships with EMT-related programs and the tumor immune microenvironment warrant further mechanistic investigation. These findings suggest that SLC35D1 may have prognostic relevance in CRC, although its prognostic value requires further validation in independent clinical cohorts.

## 1. Introduction

Colorectal cancer (CRC) is the third-most common cancer in men and the second-most common cancer in women worldwide, and it is the most common cancer with an increasing incidence rate over the years in Taiwan [1,2]. Previous studies have highlighted that the development of CRC is a complex multistep process involving the interplay of genetic and environmental factors [3]. Common genetic mutations and genomic instability have been associated with CRC [4]. The current treatment modalities for CRC primarily involve surgery, chemotherapy, and radiation therapy [5]. In recent years, targeted therapies and immunotherapies have emerged as promising treatment options [6]. However, the five-year survival rate for patients with metastatic CRC remains below 20% [7]. Growing evidence underscores the potential of molecular biomarkers for prognostic prediction and guiding treatment selection. However, many fail to advance to clinical application due to insufficient validation or limited sensitivity and specificity [8]. Therefore, to enhance biomarker-driven personalized treatment, it is crucial to identify more reliable molecular biomarkers involved in the malignant progression of CRC.

With over 400 members, solute carriers (SLCs) are the human genome’s largest family of transmembrane transporters. Although SLCs mediate essential biological functions, and several are important pharmacological targets, a significant proportion of them are poorly characterized and not assigned a substrate [9]. In addition, the dysregulation of SLCs is associated with many human diseases, such as altered metabolic programming in tumor cells leading to changes in the demand for carbohydrates, proteins, lipids, and nucleic acids. The expression of SLCs, the primary transporters for these macromolecules, is also altered accordingly. Therefore, modulating SLCs represents a potential therapeutic strategy [10]. The SLC35 family, comprising at least 25 genes, is divided into seven subfamilies, including SLC35A1-5, SLC35B1-4, SLC35C1-2, SLC35D1-3, SLC35E1-4, SLC35F1-6, and SLC35G1-4 [11]. The function of the SLC35 family is to transport nucleotide sugars, and these transporters play a crucial role in connecting the synthesis of activated sugars occurring in the nucleus or cytosol with the glycosyltransferases located within the lumen of the endoplasmic reticulum (ER) and/or Golgi apparatus [12].

The SLC35D family consists of three members, namely SLC35D1-3. SLC35D1 is a gene that encodes an endoplasmic reticulum nucleotide-sugar transporter (NST), which plays a vital role in transporting essential substrates involved in chondroitin sulfate biosynthesis [13]. In particular, SLC35D1 is responsible for facilitating the transport of UDP-glucuronic acid (UDP-GlcUA) and UDP-N-acetyl-α-D-galactosamine (UDP-GalNAc), which are critical for synthesizing chondroitin sulfate disaccharide repeats [14]. SLC35D2 encodes a Golgi-localized nucleotide-sugar transporter. Heterologous expression studies have identified UDP-N-acetyl-α-D-glucosamine (UDP-GlcNAc) as a candidate substrate for SLC35D2 [15,16]. In contrast, SLC35D1 is an endoplasmic reticulum-localized transporter that transports UDP-glucuronic acid and UDP-N-acetyl-α-D-galactosamine [14]. SLC35D3 has been reported to have a role in adipose deposition and metabolic control in addition to its function as a nucleotide-sugar transporter [17]. Furthermore, SLC35D3 promotes the formation of the BECN1-ATG14-PIK3C3 complex to induce autophagy [18]. In summary, exploring whether the SLC35D family possesses physiological functions and biological roles in tumors beyond its established NST activity presents an intriguing area of research.

Beyond its established function as a nucleotide-sugar transporter involved in chondroitin sulfate biosynthesis, SLC35D1 provides substrates required for the synthesis of chondroitin sulfate glycosaminoglycan (GAG) chains, which are incorporated into cell-surface and extracellular matrix proteoglycans. Alterations in GAG biosynthesis and composition can influence extracellular matrix (ECM) organization and interactions with cell-surface receptors, cytokines, and growth factors [19,20]. Notably, altered chondroitin sulfate abundance and composition within the tumor microenvironment have been implicated in CRC progression, including EMT-related changes and therapeutic response [21]. More broadly, remodeling of ECM glycosaminoglycans and proteoglycans has been associated with EMT, invasive behavior, and metastatic progression across multiple solid tumor types [22]. These observations provide a biological rationale for investigating whether altered SLC35D1 expression is associated with CRC progression through changes in GAG/ECM-related processes. However, the role of SLC35D1 in CRC and the molecular mechanisms underlying these potential associations remain largely unexplored.

In this study, we systematically investigated the expression and clinical relevance of the SLC35D family in CRC and further characterized SLC35D1 using bioinformatic and in vitro approaches. SLC35D1 was downregulated in CRC and was associated with clinical outcome in the TCGA cohort. Transcriptomic analyses identified associations between reduced SLC35D1 expression and EMT- and inflammation-related programs, while experimental manipulation of SLC35D1 altered CRC cell proliferation, migration, invasion, and selected EMT-related molecular phenotypes. These findings provide a basis for further investigation of the biological and potential clinical relevance of SLC35D1 in CRC.

## 2. Results

### 2.1. SLC35D Family Genes Are Altered in CRC Patients

Using the GENT2 database, we analyzed the expression levels of SLC35D family genes across various cancers (Figure S1). SLC35D1 was significantly downregulated in several cancers, including colon, kidney, and liver, but upregulated in brain and lung cancers. SLC35D2 showed similar trends with elevated levels in breast, prostate, and liver cancers, while SLC35D3 was generally upregulated in tumors, notably colon cancer, but downregulated in adrenal gland and tongue cancers. Additionally, the analysis of the TCGA-COADREAD dataset revealed differential expression of SLC35D1, D2, and D3 in colorectal tumors compared to normal tissues. SLC35D1 was significantly downregulated in CRC tissues (*p* < 0.001), SLC35D3 was markedly upregulated (*p* < 0.001), and SLC35D2 showed a non-significant reduction (Figure 1A). These findings were consistent across TCGA and GENT2 databases. Further analysis of TCGA COADREAD data comparing early-stage (I and II) and advanced-stage (III and IV) CRC showed that SLC35D1 expression was significantly lower in advanced stages, while SLC35D3 expression was significantly higher. No significant changes were observed for SLC35D2 (Figure 1B). These results demonstrate distinct expression patterns of SLC35D family members in CRC and suggest that reduced SLC35D1 expression may be associated with disease progression.

### 2.2. Genetic Alterations of SLC35D Family in CRC Patients

It is well-established that genetic mutations drive cancer development. Using cBioPortal, we analyzed the mutation rates of SLC35D genes in CRC. SLC35D1 exhibited a mutation rate of 1.4%, primarily involving missense and truncating mutations. SLC35D2 had the highest mutation frequency at 1.8%, including missense mutations and amplifications. SLC35D3 showed the lowest rate at 0.9%, with only missense mutations (Figure S2A). All identified genomic alterations, with their amino acid changes and predicted functional consequences, are listed in Table S1. Eight somatic mutations were detected across seven patients, together with one SLC35D2 amplification; the truncating variant noted above was the SLC35D1 nonsense mutation p.Gly14Ter (exon 1 of 12), which introduced a premature termination codon and may trigger nonsense-mediated decay, potentially reducing functional SLC35D1 dosage, whereas the remaining variants were missense substitutions of variable predicted severity. Sex-stratified alteration frequencies were given in Table S2 and did not differ significantly between male and female patients for SLC35D1 (2/116, 1.72% versus 1/107, 0.93%), SLC35D2 (2/116, 1.72% versus 2/107, 1.87%), SLC35D3 (2/116, 1.72% versus 0/107, 0%), or for any SLC35D family gene (5/116, 4.31% versus 3/107, 2.80%; Fisher’s exact test, *p* = 0.723). Given that fewer than ten patients carried an alteration, this comparison is underpowered and should be regarded as exploratory data. Correlation analysis demonstrated that the expression levels of SLC35D1, SLC35D2, and SLC35D3 were not significantly correlated with each other (Figure S2B), suggesting that their expression patterns may be regulated independently in CRC.

### 2.3. Clinical Impact of SLC35D Family Genes in CRC Patients

Kaplan–Meier analysis in the TCGA COADREAD cohort and Kaplan–Meier Plotter datasets showed that lower SLC35D1 expression was associated with shorter overall survival (*p* = 0.029 and 0.0023, respectively; Figure 2A,B), whereas SLC35D2 and SLC35D3 expression showed no significant association with survival. PrognoScan analysis further identified significant associations between SLC35D1 expression and survival outcomes in several CRC datasets (Figure 2C). In the TCGA COADREAD cohort, multivariate Cox regression analysis showed that SLC35D1 expression remained significantly associated with overall survival after adjustment for available clinicopathological variables (HR = 0.743, *p* = 0.021; Table 1). These findings support a prognostic association of SLC35D1 within the TCGA and selected public datasets; however, as described below, this association was not reproduced in the independent GSE39582 cohort.

### 2.4. SLC35D1 Expression Is Associated with Clinicopathological Features in CRC

To investigate the association between clinical features of CRC and SLC35D1, CRC patients were categorized into low (*n*  = 322) and high (*n*  =  322) SLC35D1 expression groups based on their relative expression levels. Analysis of clinicopathological features (Table 2) showed that low SLC35D1 expression was significantly associated with advanced disease stages, including stage III & IV (*p* = 0.019), N1 & N2 stage (*p* = 0.015), and lymphatic invasion (*p* = 0.001). These findings demonstrate an association between lower SLC35D1 expression and adverse clinicopathological characteristics in the TCGA COADREAD cohort.

### 2.5. Reduced SLC35D1 Expression Is Associated with EMT-Related Transcriptional Programs in Colorectal Cancer

The downregulation of SLC35D1 in CRC and its association with advanced tumor stages prompted us to investigate the role of SLC35D1 in CRC. We used Gene Set Enrichment Analysis to explore the potential biological pathways associated with SLC35D1 in CRC. This analysis was performed to identify enriched pathways based on the differential expression profiles between the low and high SLC35D1 expression groups. The low-expression group was used as the reference group to analyze the DEGs between the two groups (Figure 3A). GSEA analysis was performed based on the fold change in DEGs (Table S3). A negative normalized enrichment score (NES) indicates pathway enrichment in the SLC35D1-low group. GSEA analysis showed significant enrichment of the Kras Signaling Dn, Coagulation, Complement, EMT, IL6/JAK/STAT3 Signaling, and other immune-related pathways, indicating that the genes driving these enrichments are upregulated in SLC35D1-low tumors (Figure 3B). Among the enriched pathways, the epithelial–mesenchymal transition (EMT) signature ranked among the most significantly enriched pathways in SLC35D1-low tumors (Figure 3C), supporting an association between reduced SLC35D1 expression and EMT-related phenotypes in colorectal cancer. To assess whether the biological characteristics of SLC35D1 in CRC exist outside of TCGA, we performed the corresponding analysis in GSE39582, an independent cohort of 566 primary colorectal tumors profiled on the Affymetrix Human Genome U133 Plus 2.0 array. Consistent with the TCGA cohort, SLC35D1 expression was also significantly lower in primary CRC tumors than in non-tumoral mucosa in GSE39582 (Figure S3A). Tumors were dichotomized at the median SLC35D1 expression (*n* = 283 per group). At this median cutoff, SLC35D1 expression was not significantly associated with overall survival (high vs. low: HR = 1.19, 95% CI = 0.89–1.58; log-rank *p* = 0.24; Figure S3B) or recurrence-free survival (HR = 0.93, 95% CI = 0.69–1.25; log-rank *p* = 0.63; Figure S3C). Consistently, when SLC35D1 expression was analyzed as a continuous variable, Cox regression also showed no significant association with overall survival (HR = 0.91, 95% CI = 0.69–1.19, *p* = 0.50). Further, after dichotomizing at the median expression of SLC35D1, the resulting ranked gene list was analyzed against the Hallmark collection. Pathway-level analysis independently reproduced several SLC35D1-associated transcriptional signatures identified in TCGA, including enrichment of the Hallmark EMT gene set in the SLC35D1-low group (Figure S3D). These results show that the enrichment of EMT and inflammatory/coagulation programs in SLC35D1-low tumors represents a reproducible association, observed independently in two patient cohorts. To further characterize the transcriptomic programs associated with SLC35D1 expression in colorectal cancer, Gene Set Variation Analysis (GSVA) was performed to evaluate EMT-related pathway activity in TCGA COAD samples. SLC35D1 expression showed a modest but statistically significant negative correlation with EMT pathway scores (correlation coefficient = −0.111, *p* = 1.78 × 10^−2^), suggesting that reduced SLC35D1 expression is associated with enhanced EMT-related transcriptional programs in CRC (Figure 3D). Moreover, a protein–protein interaction network of DEGs was constructed using the STRING database, and 26 hub genes were identified through CytoNCA (Figure 3E, Table S4). The altered expression profiles of these hub genes identified a set of candidate genes associated with the SLC35D1-related transcriptional network (Figure 3F). These computationally identified candidates require further experimental validation. Collectively, these findings support a reproducible association between reduced SLC35D1 expression and EMT-related transcriptional programs in CRC, although the underlying molecular mechanisms remain to be determined.

### 2.6. SLC35D1 Expression Is Associated with EMT Marker Changes and Altered Cell Proliferation, Migration, and Invasion

To investigate the role of SLC35D1 in CRC, we silenced its expression in DLD-1 and HCT116 cells using shRNA. The SRB assay showed significantly increased proliferation in SLC35D1 knockdown cells compared to control cells after 48 h, while overexpression of SLC35D1 suppressed cell proliferation (Figure 4A). Additionally, silencing SLC35D1 significantly enhanced cell migration and invasion, whereas SLC35D1 overexpression significantly reduced these abilities (Figure 4B,C). These observations suggest involvement of SLC35D1 in processes critical to cancer progression. Given the association of EMT-related processes with cancer cell invasion and metastasis, we next examined EMT-related molecular changes following SLC35D1 knockdown and overexpression. Knockdown of SLC35D1 upregulated mesenchymal markers, including N-cadherin, vimentin, fibronectin, ZEB1, SNAIL, MMP1, MMP9, MMP13, and FLT1. E-cadherin, an epithelial marker critical for cell–cell adhesion, typically localizes to the cell membrane, and its downregulation is associated with malignant phenotypes and cancer progression [23]. To further examine the association between SLC35D1 expression and EMT-related phenotypes, we examined the cellular localization of E-cadherin using immunofluorescence analysis. SLC35D1 knockdown was accompanied by altered E-cadherin staining, including reduced membrane-associated staining and increased intracellular staining in DLD-1 cells (Figure 4D) [24]. On the other hand, SLC35D1 overexpression resulted in increased E-cadherin mRNA levels while downregulating mesenchymal markers, including N-cadherin, vimentin, and fibronectin. These changes in EMT marker expression were also observed in HCT116 cells (Figure 4E). To further evaluate EMT-associated changes at the protein level, Western blot analysis showed that SLC35D1 knockdown increased the levels of the mesenchymal markers N-cadherin, vimentin, and fibronectin while decreasing E-cadherin expression (Figure 4F). Conversely, SLC35D1 overexpression produced the opposite pattern (Figure 4G). Collectively, these findings indicate that SLC35D1 knockdown is associated with a more mesenchymal-like marker profile and increased proliferative, migratory, and invasive capacities in colorectal cancer cells, supporting an association between SLC35D1 and EMT-related processes.

### 2.7. SLC35D1 Knockdown Is Associated with Increased Stemness-Related Phenotypes in CRC Cells

Metastasis remains a major challenge in cancer treatment, with cancer stem cells (CSCs) from primary tumors playing a critical role. Colony and sphere formation assays were performed to examine stemness-related phenotypes following SLC35D1 knockdown in CRC cells. Knockdown of SLC35D1 significantly increased the size and number of colonies and spheres (Figure 5A,B). Additionally, CSC-related genes, including CD44, STAT3, LGR5, OCT4, C-MYC, and SOX2, were upregulated following SLC35D1 silencing (Figure 5C). Collectively, these findings indicate that SLC35D1 knockdown is associated with increased colony- and sphere-forming capacity and altered expression of selected stemness-related markers in DLD-1 cells.

### 2.8. In Silico Association Between SLC35D1 Expression and Estimated Immune Infiltration in CRC

Tumor-infiltrating lymphocytes play a significant role in improving cancer prognosis [25]. Given the clinical associations observed for SLC35D1 in the TCGA cohort, its relationship with estimated immune infiltration in CRC was further investigated using the TIMER database. Analysis revealed that SLC35D1 expression positively correlated with the infiltration of B cells (*p* < 0.01, r = 0.239), CD8^+^ T cells (*p* < 0.01, r = 0.208), CD4^+^ T cells (*p* < 0.01, r = 0.211), macrophages (*p* < 0.05, r = 0.105), and dendritic cells (*p* < 0.01, r = 0.153) (Figure 6A). Scatter plots based on TCGA COAD data showed a negative correlation between SLC35D1 expression and immune checkpoint markers, including PDCD1, LAG3, and HAVCR2 (Figure 6B). These findings suggest that SLC35D1 expression is associated with patterns of immune cell infiltration and immune checkpoint expression in CRC. However, the causal role for SLC35D1 in shaping the tumor immune microenvironment remains to be determined.

## 3. Discussion

In this study, we identified distinct expression patterns within the SLC35D gene family in CRC, with SLC35D1 significantly downregulated, SLC35D2 showing a nonsignificant downward trend, and SLC35D3 significantly upregulated in tumor tissues. Such divergence is consistent with the functional heterogeneity observed among members of the same gene family in cancer [26]. Differential transcriptional regulation and epigenetic modifications may contribute to these distinct expression patterns [27,28]. In addition, SLC35D family members differ in their substrate preferences, subcellular localization, and reported biological functions, suggesting that they may not necessarily be coordinately regulated during tumor progression. Further studies are required to determine the mechanisms responsible for the differential dysregulation of SLC35D family members in CRC.

Among the SLC35D family members, analysis of the TCGA-COADREAD dataset revealed that SLC35D1 showed the most consistent association with clinical outcome, with lower SLC35D1 expression associated with poorer survival in patients with CRC. Univariate and multivariate Cox regression analyses further showed that SLC35D1 expression remained significantly associated with overall survival in the TCGA cohort, and lower SLC35D1 expression was associated with lymphatic invasion and advanced disease stage. GSEA indicated that lower SLC35D1 expression was associated with enrichment of EMT-related gene signatures. Consistent with these bioinformatic observations, our in vitro loss- and gain-of-function experiments showed that modulation of SLC35D1 expression was accompanied by corresponding changes in CRC cell proliferation, migration, invasion, and EMT-related molecular phenotypes. Collectively, these findings support further investigation of SLC35D1 as a candidate prognostic biomarker in CRC while recognizing that its potential clinical utility requires additional validation.

Reliable prognostic biomarkers remain important for improving risk stratification and personalized management of CRC. Approximately 20% of patients are diagnosed with metastatic disease, while an additional 25% of patients with initially localized disease eventually develop metastasis [7]. Although several molecular biomarkers have been proposed, many have not progressed to routine clinical application because of insufficient validation or limited sensitivity and specificity [29]. Our findings indicate that lower SLC35D1 expression is associated with adverse clinicopathological characteristics and poorer clinical outcomes in the TCGA cohort, suggesting potential prognostic relevance that requires further independent validation. Although the reduced expression of SLC35D1 in CRC was independently reproduced in GSE39582, its association with survival was not. This discrepancy suggests that the prognostic relevance of SLC35D1 may be cohort-dependent. Additional validation in independent clinical populations and prospective studies will therefore be required before the prognostic utility of SLC35D1 can be established.

Epithelial–mesenchymal transition is an important biological process associated with tumor invasion, metastasis, and therapeutic resistance in CRC [30]. In the present study, transcriptomic analyses revealed that reduced SLC35D1 expression was associated with enrichment of EMT-related gene signatures in TCGA COAD samples. Although the correlation between SLC35D1 expression and the EMT-related signature was statistically significant, its magnitude was weak and therefore should not be interpreted as evidence of a strong relationship or biological causality. Instead, this bioinformatic observation was considered hypothesis-generating. Consistently, experimental modulation of SLC35D1 expression altered migratory and invasive abilities and was accompanied by changes in selected EMT-related markers in CRC cells, providing additional evidence for an association between SLC35D1 and EMT-related phenotypes.

SLC35D1 has been reported to function as a nucleotide-sugar transporter involved in chondroitin sulfate biosynthesis [14]. Alterations in glycosaminoglycan composition and extracellular matrix remodeling have been implicated in EMT regulation and tumor progression in multiple cancers [31]. Therefore, altered chondroitin sulfate biosynthesis represents one possible biological link between SLC35D1 expression and the EMT-related phenotypes observed in this study. However, we did not directly examine chondroitin sulfate synthesis or extracellular matrix composition, and this possibility remains to be experimentally investigated. The same biological context may also help explain the concomitant enrichment of the coagulation hallmark observed in our transcriptomic analysis. The leading-edge genes underlying this enrichment consisted predominantly of secreted proteases and protease inhibitors, including matrix metalloproteinases, TIMP1, cathepsins, and serpins, and substantially overlapped with genes contributing to the EMT and complement signatures rather than representing canonical coagulation factors. Thus, this enrichment may reflect an extracellular matrix remodeling-associated transcriptional program rather than activation of the coagulation cascade itself. Importantly, these analyses were derived from cross-sectional transcriptomic data and are associative; whether reduced SLC35D1 expression contributes to these transcriptional programs or occurs as a consequence of them cannot be determined from the present study.

The SLC superfamily is widely expressed and plays essential physiological roles in transporting diverse substrates, including ions, nutrients, and metabolites. Dysfunction of SLC transporters has been implicated in several human diseases, including diabetes, cancer, and central nervous system disorders, making them an important but still incompletely characterized class of potential therapeutic targets [32,33]. Altered expression of SLC transporters in tumors can influence cancer cell metabolism and drug sensitivity [34,35]. Within the SLC35D family, SLC35D1 and SLC35D2 have been associated with glycosaminoglycan biosynthesis [36], and glycosaminoglycans are known to participate in tumor progression [19]. Chondroitin sulfate and other glycosaminoglycans can influence tumor–matrix interactions, EMT-related processes, and immune cell migration [20]. However, these biological effects are context-dependent and may vary according to tumor type, glycosaminoglycan abundance, chain structure, and degradation products.

The opposing expression patterns observed among SLC35D family members may therefore reflect their functional specialization rather than coordinated regulation as a single transporter family. SLC35D1, SLC35D2, and SLC35D3 differ in their tissue distributions and subcellular localization: SLC35D1 localizes predominantly to the endoplasmic reticulum, SLC35D2 to the Golgi apparatus, and SLC35D3 to early endosomal compartments [36]. In addition, combinations involving SLC35D3 and other biomarkers have been proposed for estimating colon tumor burden and aggressiveness and stratifying recurrence risk [37]. Together with gene-specific transcriptional and epigenetic regulation [27,28], these functional differences may contribute to the contrasting expression patterns observed in CRC. Nevertheless, the biological consequences of SLC35D3 upregulation and its potential relationship with SLC35D1 remain to be experimentally determined.

Previous studies have also implicated other SLC family members in tumor progression. For example, upregulation of SLC34A2 and downregulation of SLC4A4 have been associated with metastasis of papillary thyroid carcinoma [38], whereas SLC2A3 has been reported to promote CRC progression through EMT-related processes [39]. Several SLCs, including SLC2A1, SLC4A4, and SLC39A6, have also been associated with invasion and metastasis in pancreatic cancer [40]. In addition, SLC22A10 and SLC22A15 have been implicated in pancreatic cancer aggressiveness through ROR1 expression and IFN-related signaling [41]. These observations illustrate the diverse and context-dependent roles of SLC transporters in cancer biology.

The tumor immune microenvironment is an important determinant of CRC progression, treatment response, and clinical outcome [25]. SLC transporters may also influence immune cell metabolism and effector functions within the tumor microenvironment [42,43]. In the present study, bioinformatic analyses revealed associations between SLC35D1 expression, immune cell infiltration, and immune checkpoint markers. These findings suggest a potential relationship between SLC35D1 expression and the immune characteristics of CRC; however, they are based entirely on computational correlations and do not demonstrate that SLC35D1 directly regulates immune cell recruitment, activity, or immune checkpoint expression. Further experimental studies will therefore be required to determine the biological significance of these associations.

To our knowledge, the relationship between SLC35D1 and CRC has not previously been systematically investigated. Our findings identify associations between reduced SLC35D1 expression and adverse clinical characteristics and show that experimental modulation of SLC35D1 expression alters malignant phenotypes in CRC cells. Nevertheless, several limitations should be acknowledged. First, our functional evidence was derived primarily from bioinformatic analyses and in vitro experiments, and the involvement of SLC35D1 in CRC progression and EMT-related phenotypes was not validated in animal models or clinical specimens. Therefore, the associations reported in this study should not be interpreted as establishing biological causality. Second, the tumor–immune relationships suggested by TIMER-based analyses are correlative and were not experimentally tested. Immune cell co-culture systems or humanized xenograft models will be required to determine whether SLC35D1 expression in tumor cells directly influences immune cell recruitment, activation, or checkpoint expression. Third, although SLC35D1 may have potential prognostic relevance, its clinical utility and therapeutic relevance were not directly evaluated in the present study and require independent clinical validation and appropriate therapeutic studies. Finally, the molecular mechanisms underlying the association between SLC35D1 and EMT- and inflammation-related transcriptional programs remain undefined. The pathway-enrichment results should therefore be considered hypothesis-generating rather than evidence of pathway dependency. Future studies examining potential crosstalk between SLC35D1, KRAS-driven signaling, and the IL6-JAK-STAT3 axis may help clarify the molecular mechanisms linking SLC35D1 to EMT- and immune-related phenotypes in CRC.

## 4. Materials and Methods

### 4.1. Analysis of Expression Levels

SLC35D family mRNA expression of tumor and adjacent normal tissues were analyzed by GENT2 (http://gent2.appex.kr/gent2/; accessed on 10 September 2025) [44]. We navigated to the “Search—Gene profile” page, selected “Tissue” under the “Types” category, and chose “Gene symbol” under “Terms” to perform gene expression analysis. Furthermore, we retrieved the data processed through the STAR workflow from The Cancer Genome Atlas (TCGA) (https://portal.gdc.cancer.gov; accessed on 10 September 2025). The TPM data of COADREAD were normalized using the log2 transformation with the addition of 1. We compared the expression of the SLC35D family between tumor and normal tissues, as well as investigated the expression across different tumor stages. Furthermore, the expression correlations among individual SLC35D family members were examined. The statistical analysis was performed using the R packages stats [4.2.1] and car [3.1-0] by conducting the Wilcoxon rank sum test. The results were visualized using the ggplot2 package [3.3.6]. We also conducted a chi-square test to analyze the association between SLC35D1 gene expression and the clinical characteristics of the COADREAD patients.

### 4.2. Analysis of Genomic Alterations

The mutation status of the SLC35D family genes was investigated using the OncoPrint analysis function available in the cBioPortal (http://cbioportal.org; accessed on 10 September 2025) for Cancer Genomics [45]. We analyzed the mutation status of SLC35D family genes using the CRC dataset (Firehose Legacy) from TCGA (COADREAD_TCGA; accessed on 27 July 2026), using the 223 samples with available mutation data as the denominator. Identified variants were annotated with the Ensembl Variant Effect Predictor (VEP) against genome build GRCh37/hg19, including HGVS nomenclature and the SIFT and PolyPhen-2 functional predictions implemented therein. Alteration frequencies in male (*n* = 116) and female (*n* = 107) patients were compared by Fisher’s exact test, with two-sided *p* < 0.05 considered significant.

### 4.3. Survival Analysis

To evaluate the prognostic relevance of the SLC35D gene family in colorectal cancer, we utilized the Kaplan–Meier Plotter (http://kmplot.com/analysis; accessed on 10 September 2025) with GEO-derived CRC datasets [46]. Patients were stratified into high- and low-expression groups based on the median expression level of each gene, and overall survival (OS) was selected as the clinical endpoint. The database included subjects with both genders. Log-rank *p*-values, hazard ratios (HRs), and 95% confidence intervals (CIs) were calculated accordingly. In addition, RNA-seq data (FPKM normalized) and corresponding clinical annotations for colon adenocarcinoma and rectal adenocarcinoma (COADREAD) were retrieved from TCGA via the Genomic Data Commons (GDC) portal (https://portal.gdc.cancer.gov; accessed on 10 September 2025), processed using the STAR alignment workflow. Patient survival information was curated from a publication in the Cell, which provided harmonized survival data across TCGA cohorts [47]. For survival modeling, univariate and multivariate Cox proportional hazards regression analyses, along with Kaplan–Meier survival estimates, were performed using the survival R package (v3.3.1). Visualization of survival curves and regression results was accomplished using the survminer and ggplot2 packages. To further assess the prognostic value of SLC35D1 across independent CRC datasets, we accessed the PrognoScan database (http://www.abren.net/PrognoScan/; accessed on 10 September 2025) [48], conducting a meta-analysis of gene expression and survival correlations. Statistical significance was set at a two-sided *p*-value < 0.05.

### 4.4. Functional Enrichment Analysis

We employed the DESeq2 [1.36.0] package to analyze differential gene expression between the high- and low-expression groups of SLC35D1 in TCGA COAD data [49]. Differentially expressed genes (DEGs) were identified using a threshold of adjusted *p* value < 0.05 and logFC ≥ 1 or ≤−1. Subsequently, we conducted GSEA using the clusterProfiler [4.4.4] package based on the logFC values [50]. The results of the analysis were visualized using the ggplot2 [3.3.6] package. Pathway activity in TCGA COAD samples was evaluated by single-sample gene set enrichment analysis (ssGSEA) using the GSVA package in R [51]. RNA-seq expression data were normalized using log2(TPM + 1) transformation, and pathway scores were calculated using the method = “ssgsea” parameter. Correlations between SLC35D1 expression and pathway activity scores were analyzed using Spearman correlation analysis. A *p*-value < 0.05 was considered statistically significant. In addition, a protein–protein interaction (PPI) network of the DEGs was constructed using the STRING database (https://string-db.org/; accessed on 10 September 2025), with the minimum required interaction score set to high confidence (0.700) [52]. The interaction network was downloaded in TSV format and subsequently imported into Cytoscape software (version 3.9.1) for visualization. To identify hub genes within the network, the CytoNCA plugin was applied, enabling topological analysis based on network centrality measures [53,54].

### 4.5. Independent Cohort Validation of Pathway Enrichment

The series matrix file for GSE39582 was downloaded from the Gene Expression Omnibus and used as deposited (log2 scale). SLC35D1 expression was defined as the arithmetic mean of the log2 signals of probe sets 209711_at, 209712_at and 209713_s_at, and tumors were assigned to SLC35D1-high and SLC35D1-low groups at the cohort median. Probe sets were collapsed to gene symbols using the hgu133plus2.db annotation, retaining for each gene the probe set with the highest mean expression (21,355 genes). Differential expression between groups was assessed with limma using a design matrix containing the SLC35D1 group factor, genes were ranked by the moderated t statistic, and the complete ranked list was analysed with fgsea (fgseaMultilevel) against the MSigDB Hallmark collection using a fixed random seed. Enrichment *p* values were adjusted across the 50 Hallmark gene sets by the Benjamini–Hochberg method. Recurrence-free survival was defined as the interval from surgery (or diagnosis) to recurrence or death from any cause, with non-events censored at last follow-up. Overall survival was defined as the interval from surgery (or diagnosis) to death from any cause, with surviving patients censored at last follow-up. Kaplan–Meier curves were compared between high- and low-expression groups using the log-rank test. Univariate Cox proportional hazards regression was performed to estimate hazard ratios (HR) with 95% confidence intervals for RFS/OS, treating SLC35D1 expression both as a dichotomized variable (high vs. low) and as a continuous variable (per 1-unit increase in log2 expression). All analyses were conducted using Python (lifelines package, v0.29.0), with a two-sided *p* < 0.05 considered statistically significant.

### 4.6. Cell Culture, Transfection, and Generation of Stable Cell Lines

Human CRC cells, DLD-1 (RRID: CVCL_0248) and HCT116 (RRID: CVCL_0291), were purchased from American Type Culture Collection (ATCC, Rockville, MD, USA; DLD-1: catalog no. CCL-221; HCT116: catalog no. CCL-247). DLD-1 and HCT116 cells are widely used, well-characterized CRC cell lines with differing genetic backgrounds (DLD-1: KRAS/TP53-mutant, microsatellite-instable; HCT116: KRAS-mutant, TP53-wild-type, microsatellite-instable), allowing us to test SLC35D1’s phenotypic effects across distinct genetic contexts. Cell line identities were verified via the Cellosaurus database (Database Name: Cellosaurus; https://www.cellosaurus.org; accessed on 10 September 2025). All cells were cultured in RPMI 1640 medium supplemented with 10% fetal bovine serum (FBS) (SAFC Biosciences, Lenexa, KS, USA) and 1% penicillin/streptomycin (Life Technologies, Singapore) containing 100 IU/mL of penicillin and 100 μg/mL of streptomycin at 37 °C in 5% CO_2_ in a humidified incubator. Cell lines were authenticated upon receipt and when thawing new cryopreserved stocks. Mycoplasma testing was conducted every two months to ensure culture quality. SLC35D1 knockdown (KD) models were established in CRC cells using short hairpin RNA. The SLC35D1 specific shRNA (shRNA; TRCN0000233169: target sequence CAGAGGATTGCTTCATCTGAT) targeting human SLC35D1 (NM_015139) was purchased from the National RNAi Core Facility at Academia Sinica in Taipei, Taiwan. SLC35D1 overexpression was established by transfecting cells with pCMV6-Entry-SLC35D1 (catalog number: RC203263, OriGene Technologies, Inc. Rockville, MD, USA). SLC35D1-shRNA, nontarget shRNA (catalog no. SHC002, Sigma-Aldrich, Saint Louis, MO, USA; target sequence 5′-CAACAAGATGAAGAGCACCAA-3′), SLC35D1-overexpression, and vector were transfected into CRC cells using the Neon Electroporation system (Model MPK5000; Invitrogen Life Technologies, Carlsbad, CA, USA), and stably transfected cells were selected using puromycin (catalog no. ant-pr-1; InvivoGen, San Diego, CA, USA) for 2 weeks. A comprehensive reagent list is provided in Table S5.

### 4.7. Sulforhodamine B (Srb) Assay

SLC35D1-knockdown, overexpression, and their corresponding control CRC cells were seeded into 24-well plates at an initial density of 2 × 10^4^ cells per well and allowed to adhere overnight in a humidified incubator (37 °C, 5% CO_2_). The following day, culture media was removed, and the cells were fixed by adding cold 10% (*w*/*v*) trichloroacetic acid and incubating at 4 °C for 12 h. Next, the fixed monolayers were stained with a 0.4% (*w*/*v*) SRB solution (catalog no. S1402; Sigma-Aldrich) for 30 min at room temperature. To eliminate excess, non-specific dye, the wells were washed twice with 1% acetic acid and left to air-dry overnight. The incorporated dye was then dissolved in a 10 mM Tris base solution. Finally, absorbance was recorded at 515 nm using a microplate reader (Bio-Rad Laboratories, Hercules, CA, USA). Proliferation rates were calculated as fold-changes relative to the baseline values of the control group.

### 4.8. Transwell Migration and Invasion Assays

Cell migration assays were performed using BD Falcon™ cell culture inserts, while cell invasion assays were conducted using BD BioCoat™ Matrigel^®^ Invasion Chambers pre-coated with BD Matrigel Matrix (BD Biosciences, Franklin Lakes, NJ, USA). Cells (1 × 10^5^ in 500 μL serum-free RPMI) were seeded in the upper chamber, while the lower chamber contained RPMI with 10% FBS and 1% antibiotics. After 48 h at 37 °C in a CO_2_ incubator, non-migrated cells were removed, and the remaining cells were fixed, stained with 0.1% crystal violet (catalog no. C0775; Sigma-Aldrich), and incubated at room temperature for 8 h. Stained cells on the reverse membrane were counted under a microscope (10× magnification).

### 4.9. Colony Formation Assay

Cells were seeded in 6-well plates at a density of 1 × 10^3^ cells per well and cultured in a 5% CO_2_ incubator at 37 °C. After 10 days, cells were fixed with 0.4% formaldehyde (catalog no. H121-08; Macron Fine Chemicals, Center Valley, PA, USA) and stained with 0.1% crystal violet (catalog no. C0775; Sigma-Aldrich). Colony numbers were quantified using ImageJ (v1.53).

### 4.10. Sphere Formation Assay

Cells were cultured in serum-free RPMI 1640 medium containing 10 ng/mL bFGF (catalog no. 100-18B-100UG; PeproTech, Rocky Hill, NJ, USA), 20 ng/mL EGF (catalog no. cyt-332; Prospec, Bengaluru, Karnataka), and 1× B27 supplement (catalog no. 17504044; Thermo Fisher Scientific, Waltham, MA, USA), and seeded in ultralow-attachment 6-well plates. After 14 days of incubation, the formed spheroids were photographed and counted.

### 4.11. Quantitative Reverse-Transcription Polymerase Chain Reaction

Total RNA was extracted from cultured CRC cells using TRIZOL reagent (Invitrogen Life Technologies, Carlsbad, CA, USA) following the manufacturer’s protocol. Subsequently, 8 μg of extracted RNA was reverse-transcribed into cDNA using the cDNA Synthesis Kit (Invitrogen Life Technologies) in a 20 μL reaction volume, with procedures performed strictly according to the manufacturer’s guidelines. Quantitative polymerase chain reaction was conducted using an ABI 7500 FAST Real-Time PCR System with SYBR Green Master Mix (Applied Biosystems, Waltham, MA, USA). Each PCR reaction was prepared in a total volume of 30 μL, containing 1.5 μL of cDNA template, 3 μL of primers, 12 μL of nuclease-free water, and 15 μL of SYBR Green Master Mix. After thorough mixing, 10 μL of the reaction mixture was aliquoted into each well of a 96-well plate. The thermal cycling protocol consisted of an initial denaturation step at 95 °C for 10 min, followed by 45 cycles of denaturation at 95 °C for 15 s and combined annealing/extension at 60 °C for 1 min. All samples were analyzed in triplicate within each experiment, and the entire experimental procedure was independently repeated three times to ensure reproducibility. Target gene expression levels were normalized to GAPDH.

### 4.12. Western Blot Assay

For protein extraction, 1 × 10^6^ CRC cells were seeded into 10 cm culture dishes and incubated for 48 h. Cell lysates were then prepared and subjected to sodium dodecyl sulfate–polyacrylamide gel electrophoresis (SDS-PAGE) for protein separation. Following electrophoresis, proteins were transferred onto polyvinylidene fluoride (PVDF) membranes (GE Healthcare, Piscataway, NJ, USA) for immunoblotting analysis. The membranes were incubated overnight at 4 °C with primary antibodies targeting GAPDH (catalog no. IR3-8; iReal Biotechnology, Hsinchu City, Taiwan), SLC35D1 (catalog no. GTX85390; GeneTex, Irvine, CA, USA), fibronectin (catalog no. IR48-147; iReal Biotechnology), N-cadherin (catalog no. IR46-143; iReal Biotechnology), E-cadherin (catalog no. IR55-180; iReal Biotechnology), or vimentin (catalog no. IR45-137; iReal Biotechnology). After washing, membranes were incubated with appropriate secondary antibodies (HRP-conjugated IgG, catalog no. 111-035-003; Jackson ImmunoResearch, West Grove, PA, USA) for 1 h at room temperature. Protein bands were visualized using enhanced chemiluminescence (ECL) detection reagent (GE Healthcare, Piscataway, NJ, USA), and relative protein expression levels were quantified using the VersaDoc 5000 imaging system (Bio-Rad Laboratories, Hercules, CA, USA).

### 4.13. Immune Infiltration and Immune Checkpoints Analysis

To assess the relationship between SLC35D family members and tumor-infiltrating immune cells in CRC, we utilized the TIMER (Tumor Immune Estimation Resource) database (https://cistrome.shinyapps.io/timer/; accessed on 10 September 2025), a publicly available web platform that enables systematic analysis of immune infiltrates across multiple cancer types [55]. Specifically, we employed the “Gene” module to explore the correlation between gene expression levels and the estimated abundance of six major immune cell types: B cells, CD4^+^ T cells, CD8^+^ T cells, neutrophils, macrophages, and dendritic cells. The immune infiltration levels were inferred using TIMER’s deconvolution algorithms based on RNA-seq expression profiles from TCGA. Correlation analyses were visualized as scatter plots with Spearman’s correlation coefficients, and statistical significance was assessed automatically by the platform. Next, we retrieved RNA-seq data of colon adenocarcinoma (COAD) from TCGA via the Genomic Data Commons portal (https://portal.gdc.cancer.gov; accessed on 10 September 2025). The dataset was pre-processed using the STAR alignment workflow provided by TCGA. Transcript-level expression values were transformed to transcripts per million (TPM), followed by log2(TPM + 1) normalization for downstream analyses. To investigate the relationship between the SLC35D gene family and immune checkpoint molecules, we performed Spearman’s rank correlation analysis. All correlation analyses were conducted using the ggplot2 package (version 3.3.6) in R. A two-tailed *p* value less than 0.05 was considered statistically significant.

### 4.14. Statistical Analysis

All experiments were conducted at least three times. Data from real-time RT-PCR and cell proliferation assays are presented as mean  ±  SD. Representative images are shown where indicated, with quantitative analyses derived from at least three independent biological experiments. For comparisons between two groups, statistical significance was assessed using a two-tailed Student’s *t*-test. Paired two-tailed Student’s *t*-tests were used for Western blot densitometric analyses because control and genetically manipulated samples were obtained from matched independent experiments. Asterisks in the figures denote statistically significant differences between the experimental groups and their respective controls (*p* < 0.05; refer to figure legends for details).

## 5. Conclusions

Our study identified distinct expression patterns among SLC35D family members in CRC and showed that lower SLC35D1 expression was associated with adverse clinicopathological features and poorer clinical outcomes in the TCGA cohort. Functional enrichment analyses revealed associations between reduced SLC35D1 expression and EMT-related transcriptional signatures, while loss- and gain-of-function experiments showed that SLC35D1 expression is associated with CRC cell proliferation, migration, invasion, and EMT-related molecular phenotypes. SLC35D1 expression was also associated with immune cell infiltration and immune checkpoint markers, although the biological significance of these relationships requires experimental validation. Collectively, these findings identify SLC35D1 as an understudied factor associated with CRC progression and suggest potential prognostic relevance, although its prognostic value requires further validation in independent clinical cohorts. Additional mechanistic and in vivo studies are required to determine how SLC35D1 contributes to CRC biology and whether it has therapeutic relevance.

## Figures and Tables

**Figure 1:**
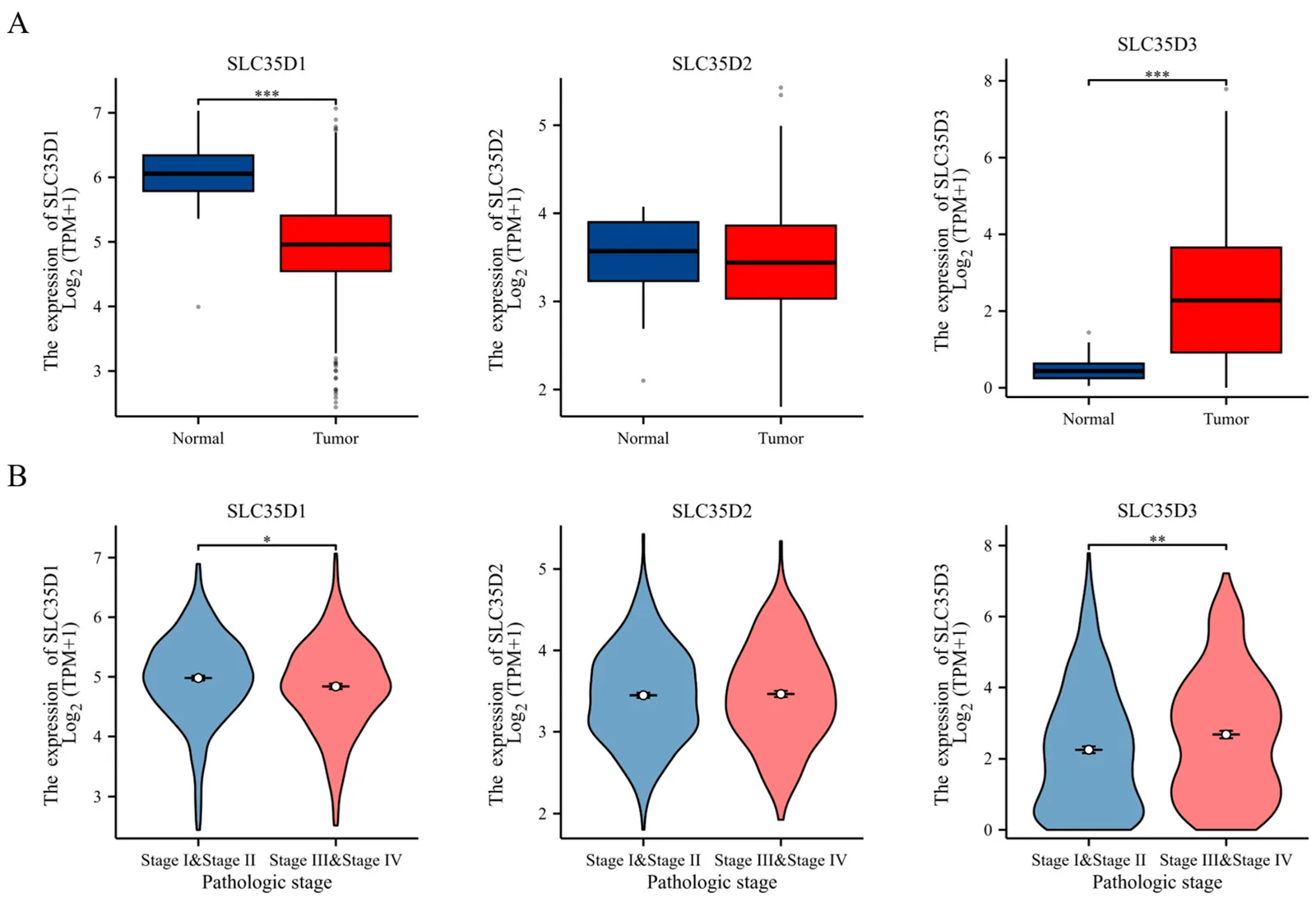
Expression patterns of SLC35D family members in human colorectal cancer. (**A**) Expression levels of SLC35D1–3 in CRC and normal tissues from the TCGA COADREAD cohort. (**B**) Association between SLC35D1–3 expression and pathological stage in patients with CRC. * *p* < 0.05, ** *p* < 0.01, and *** *p* < 0.001.

**Figure 2:**
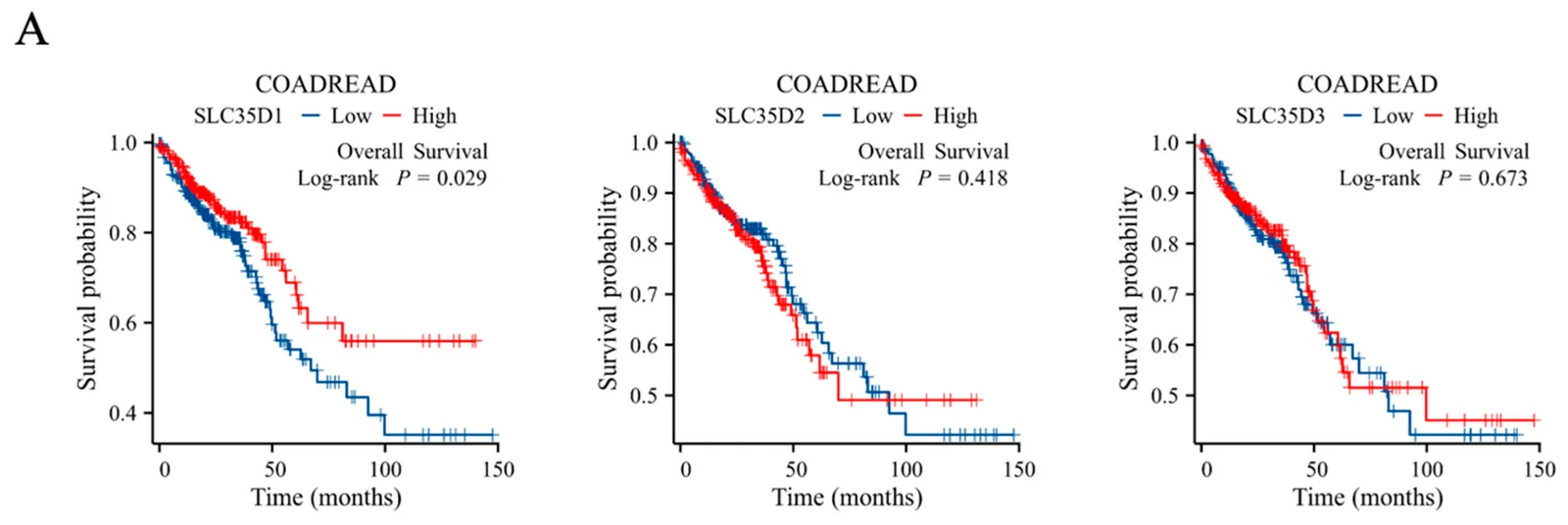

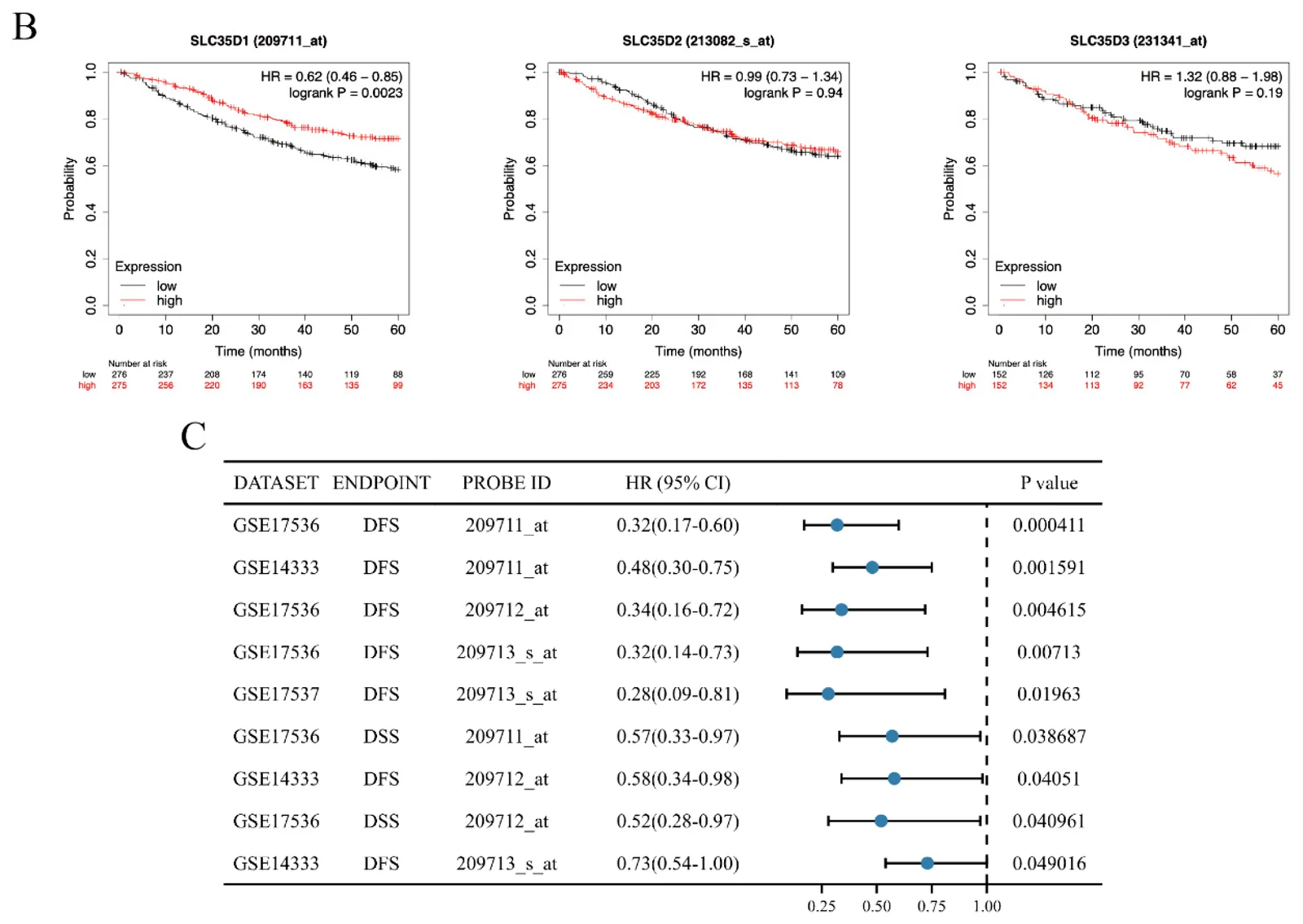
Prognostic relevance of SLC35D family gene expression in colorectal cancer. (**A**) Overall survival analysis of SLC35D family members in the TCGA COADREAD cohort. (**B**) Overall survival analysis using the Kaplan–Meier Plotter colon cancer datasets. (**C**) Cox regression analysis of the association between SLC35D1 expression and survival outcomes across CRC datasets in PrognoScan.

**Figure 3:**
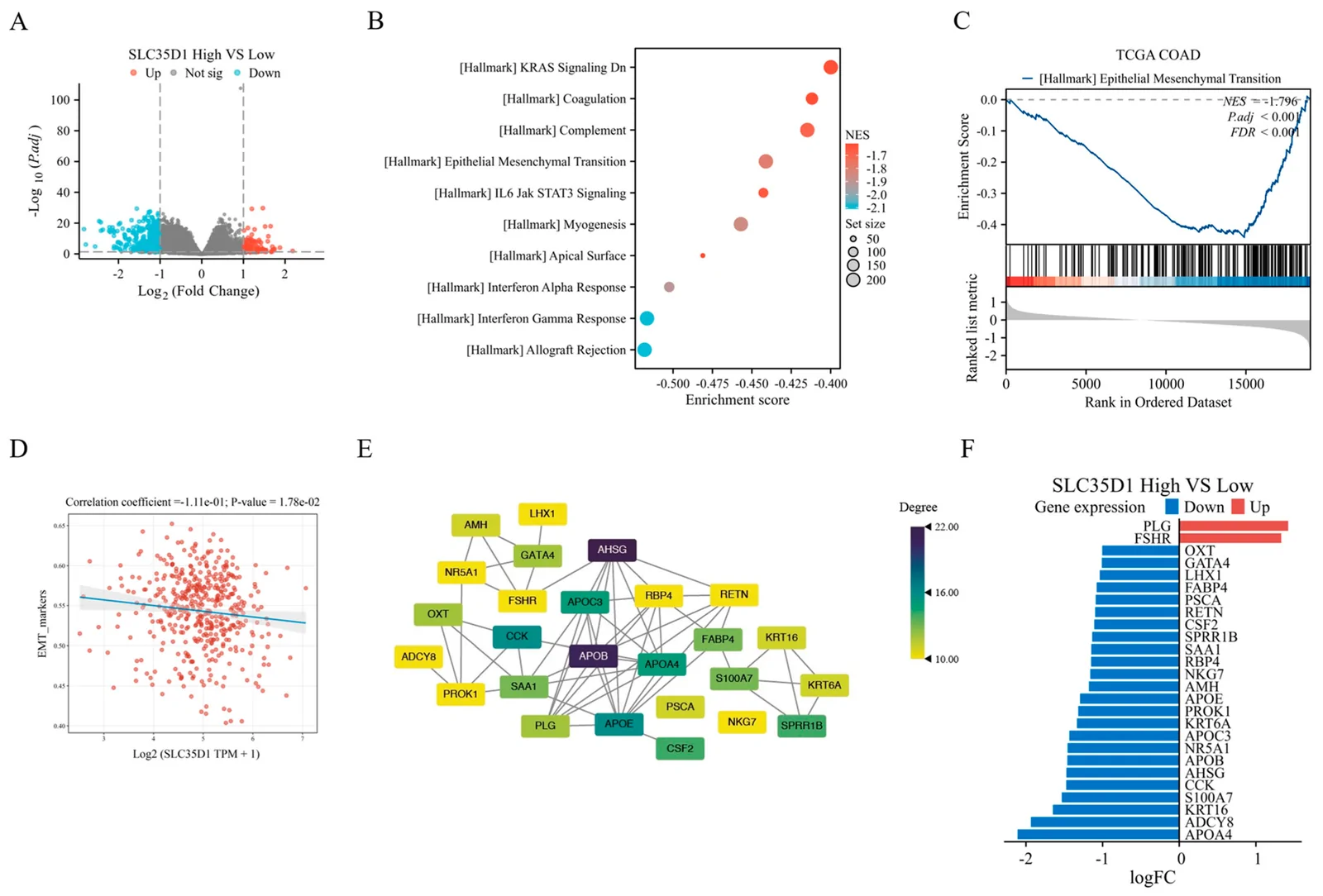
Correlation between SLC35D1 expression and EMT pathway activity score calculated by ssGSEA in TCGA COAD samples. (**A**) The volcano plot represents the differential genes between high and low groups. GSEA analysis of all DEGs using hallmark gene set collections. (**B**) Bubble chart of the GSEA results (Bubble plot showing the top 10 negatively enriched Hallmark gene sets). (**C**) GSEA enrichment plot of HALLMARK_Epithelial-Mesenchymal-Transition Signaling. (**D**) GSVA of EMT-related pathway activity. (**E**) PPI analysis and hub gene screening of SLC35D1-high and -low DEGs. (**F**) A bidirectional bar chart showing the log fold change of 26 hub genes.

**Figure 4:**
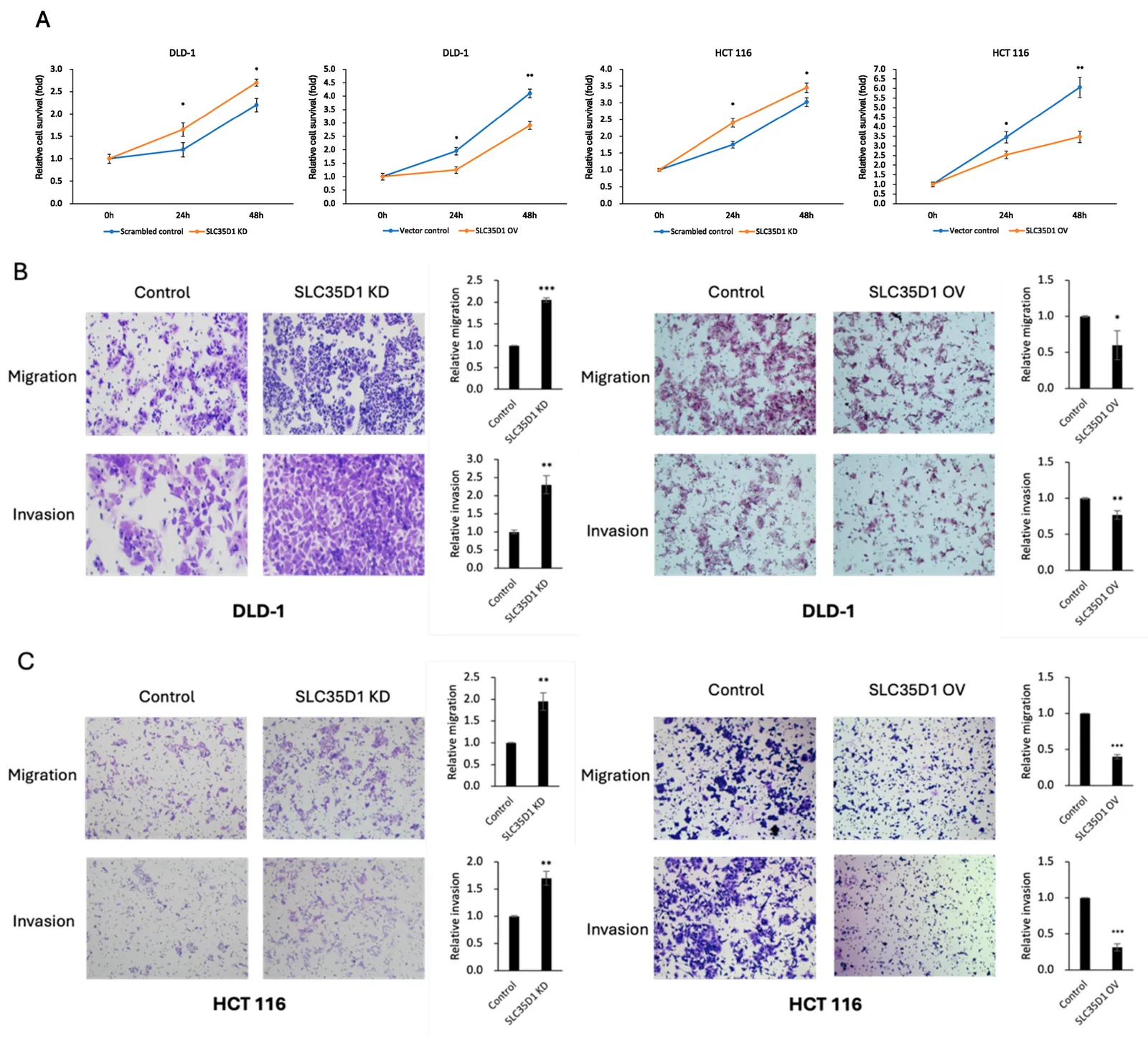

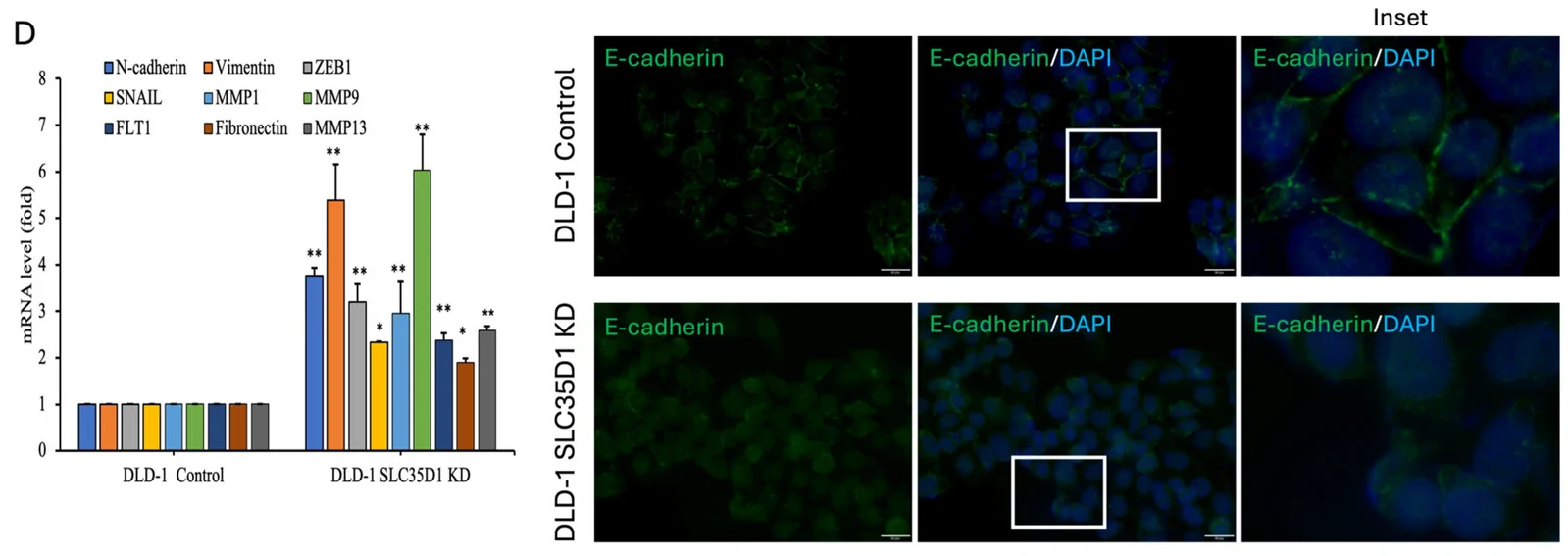

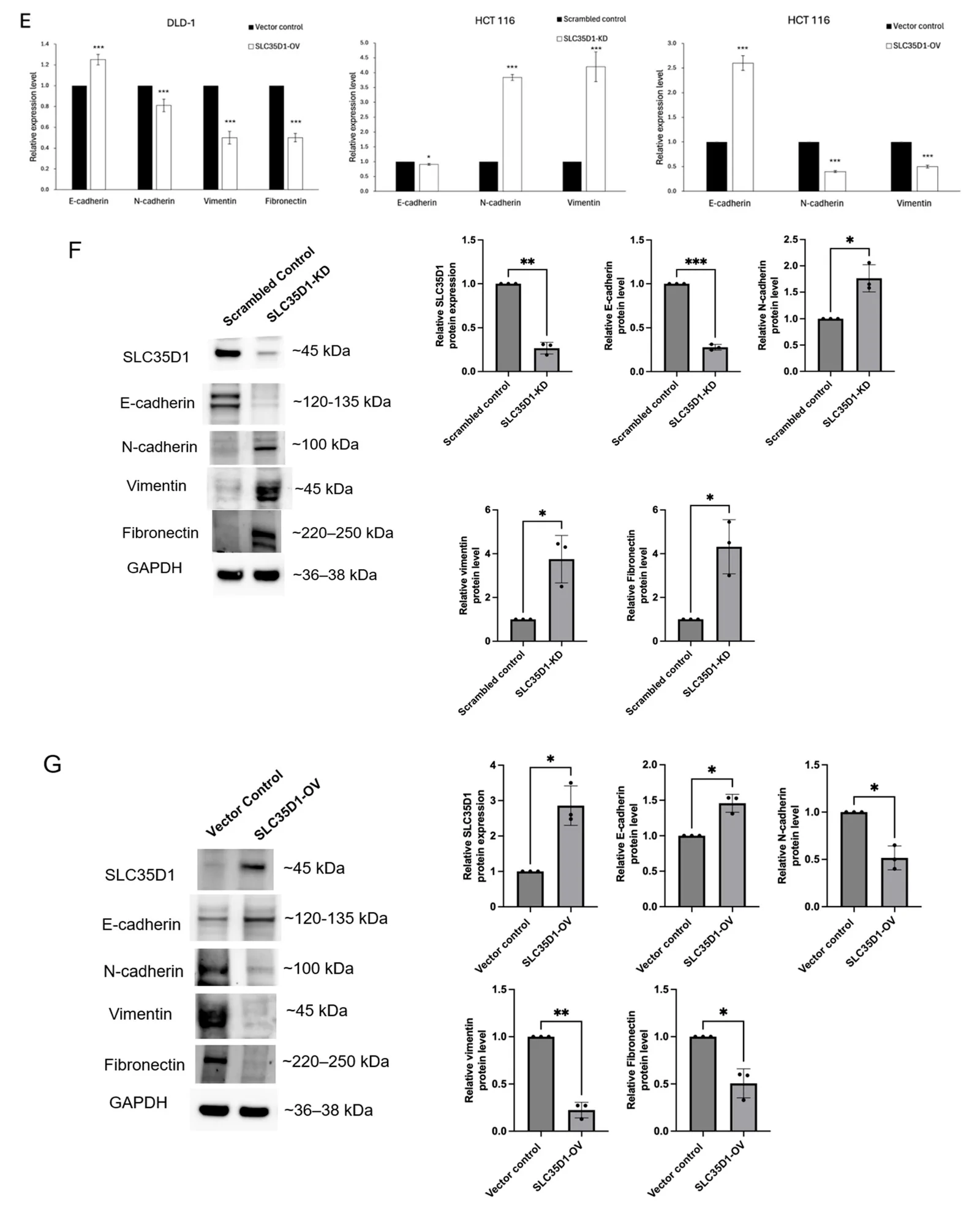
Effects of SLC35D1 knockdown and overexpression on CRC cell proliferation, migration, invasion, and EMT-related markers. (**A**–**C**) Effects of SLC35D1 knockdown and overexpression on CRC cell proliferation, migration, and invasion. (**D**) Expression of EMT-associated genes in SLC35D1-knockdown DLD-1 cells (**left**) and representative immunofluorescence images of E-cadherin localization (**right**), scale bar: 20 μm. (**E**) Expression of EMT-associated genes in SLC35D1-overexpressing DLD-1 cells and in SLC35D1-knockdown and -overexpressing HCT116 cells. (**F**) Representative Western blot images and densitometric quantification of SLC35D1 and EMT-associated proteins in SLC35D1-knockdown cells and the corresponding scrambled control cells. (**G**) Representative Western blot images and densitometric quantification of SLC35D1 and EMT-associated proteins in SLC35D1-overexpressing cells and the corresponding vector control cells. Protein expression was normalized to GAPDH and expressed relative to the corresponding control. Statistical significance for Western blot densitometric analyses was determined using a paired two-tailed Student’s *t*-test. * *p* < 0.05, ** *p* < 0.01, and *** *p* < 0.001.

**Figure 5:**
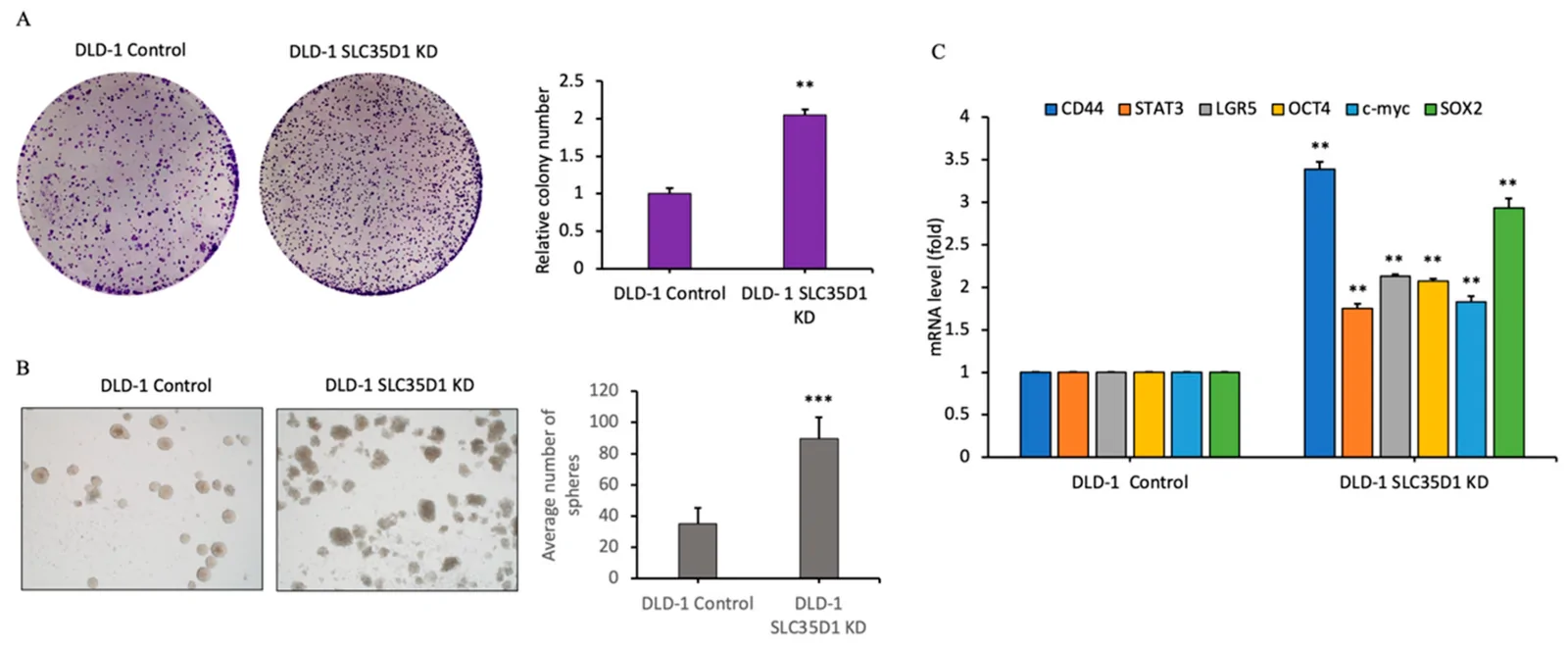
Association of SLC35D1 knockdown with stemness-related phenotypes in DLD-1 cells. (**A**) Colony formation and (**B**) sphere formation in control and SLC35D1-knockdown cells. (**C**) mRNA expression levels of selected stemness-related markers. ** *p* < 0.01, and *** *p* < 0.001.

**Figure 6:**
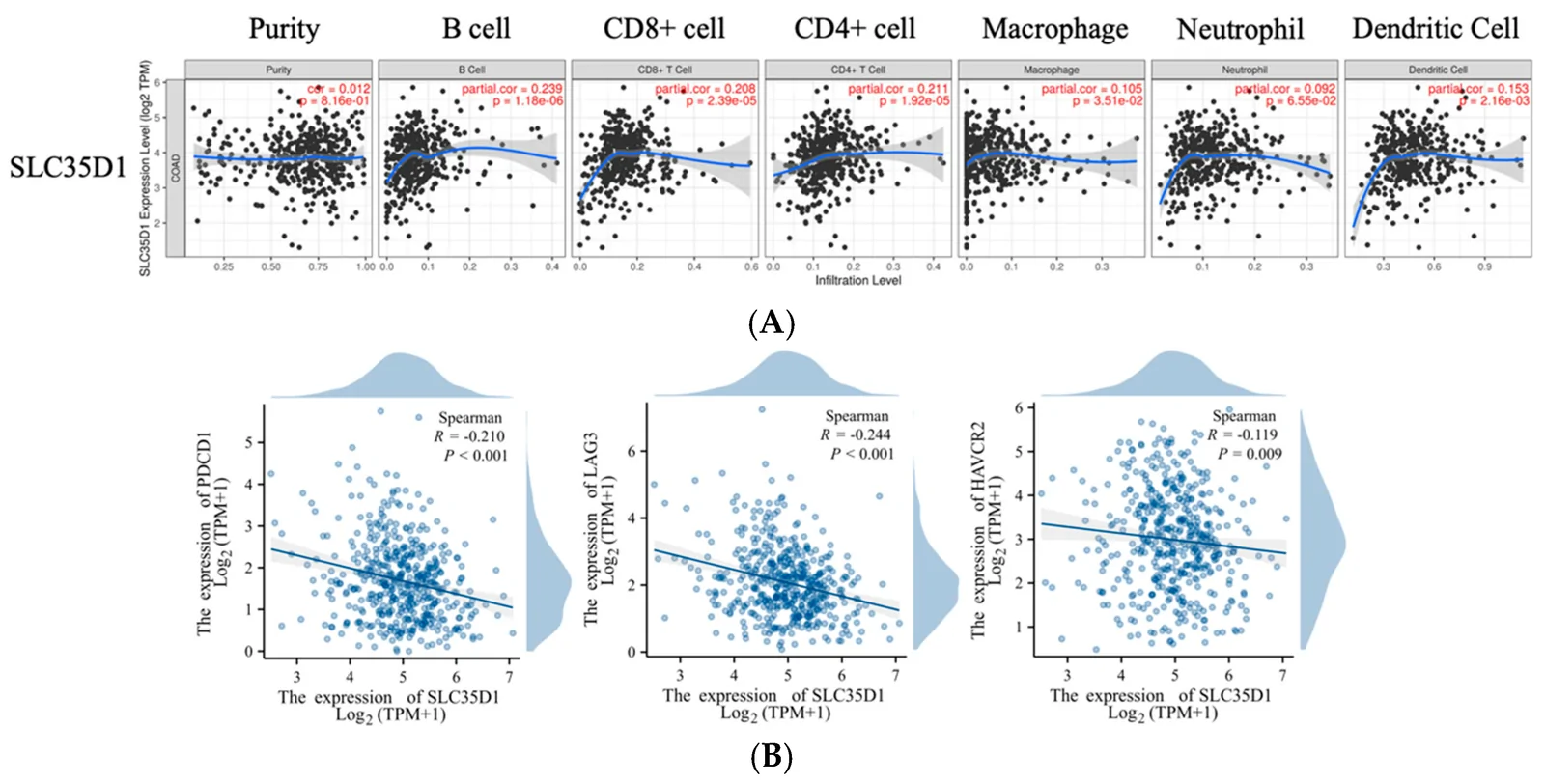
Tumor immune infiltration and immune checkpoint analysis. (**A**) Association between SLC35D1 and tumor-infiltrating immune cells in COAD. (**B**) Scatter plots visualizing a significant correlation between immune checkpoints and SLC35D1 expression.

**Table 1:** Univariate and multivariate cox regression models for overall survival (OS) in patients with COADREAD in TCGA.

Characteristics	Total (*N*)	Univariate Analysis		Multivariate Analysis	
		Hazard Ratio (95% CI)	*p* Value	Hazard Ratio (95% CI)	*p* Value
Gender	643		0.769		
Female	301	Reference			
Male	342	1.054 (0.744–1.491)	0.769		
Age	643		<0.001		
≤65	276	Reference		Reference	
>65	367	1.939 (1.320–2.849)	<0.001	2.571 (1.657–3.989)	<0.001
Pathologic stage	622		0.248		
Stage I & Stage II	295	Reference			
Stage III & Stage IV	327	1.240 (0.859–1.789)	0.251		
Pathologic T stage	640		0.001		
T1 & T2	131	Reference		Reference	
T3 & T4	509	2.468 (1.327–4.589)	0.004	2.184 (0.992–4.808)	0.052
Pathologic N stage	639		<0.001		
N0	367	Reference		Reference	
N1 & N2	272	2.627 (1.831–3.769)	<0.001	1.691 (1.060–2.698)	0.028
Pathologic M stage	563		<0.001		
M0	474	Reference		Reference	
M1	89	3.989 (2.684–5.929)	<0.001	3.040 (1.906–4.849)	<0.001
SLC35D1	643	0.713 (0.565–0.900)	0.004	0.743 (0.576–0.957)	0.021
SLC35D2	643	1.135 (0.840–1.532)	0.409		
SLC35D3	643	0.980 (0.885–1.085)	0.699		

Note. Of the 644 patients in TCGA COADREAD cohort, one patient was excluded from the Cox analysis due to lack of survival time.

**Table 2:** The relation between SLC35D1 expression and clinical characteristics in CRC (TCGA COADREAD).

Characteristics	Low Expression of SLC35D1	High Expression of SLC35D1	*p* Value
*n*	322	322	
Gender, *n* (%)			0.693
Female	153 (23.8%)	148 (23%)	
Male	169 (26.2%)	174 (27%)	
Age, *n* (%)			0.339
≤65	132 (20.5%)	144 (22.4%)	
>65	190 (29.5%)	178 (27.6%)	
Pathologic stage, *n* (%)			0.019
Stage I & Stage II	163 (26.2%)	186 (29.9%)	
Stage III & Stage IV	154 (24.7%)	120 (19.3%)	
Pathologic T stage, *n* (%)			0.182
T1 & T2	59 (9.2%)	72 (11.2%)	
T3 & T4	263 (41%)	247 (38.5%)	
Pathologic N stage, *n* (%)			0.015
N0	170 (26.6%)	198 (30.9%)	
N1 & N2	152 (23.8%)	120 (18.8%)	
Pathologic M stage, *n* (%)			0.226
M0	239 (42.4%)	236 (41.8%)	
M1	51 (9%)	38 (6.7%)	
Lymphatic invasion, *n* (%)			0.001
No	159 (27.3%)	191 (32.8%)	
Yes	137 (23.5%)	95 (16.3%)	

## Data Availability

The original contributions presented in this study are included in the article/Supplementary Materials. Further inquiries can be directed to the corresponding authors.

## Supplementary Materials

The following supporting information can be downloaded at: https://www.mdpi.com/article/10.3390/ijms27198517/s1.
