# Supplementary material for: SLC35D1 Is Associated with Colorectal Cancer Progression, EMT-Related Phenotypes, and Immune Infiltration
Source: Int J Mol Sci. 2026 Sep 24;27(19):8517. doi: 10.3390/ijms27198517 (PMC13664988; doi:10.3390/ijms27198517)
Supplement: Supplementary file 1 [file ijms-27-08517-s001.zip › Table S1.pdf]

Supplementary Table S1. Somatic alterations of SLC35D family genes in colorectal cancer (TCGA COADREAD, Firehose Legacy).

Gene	Sample ID	Sex	Chromosome	Position (GRCh37)	Reference allele	Alternate allele	Variant classification	Transcript	HGVS c.	HGVS p.	Amino acid change	Exon	Known variant ID	SIFT	PolyPhen-2	Potential implication for expression or function
SLC35D1	TCGA-AG-A002-01	Male	1	67519657	C	A	Nonsense Mutation	ENST00000235345	c.40G>T	p.Gly14Ter	p.G14*	1/12	novel	n/a	n/a	Premature termination codon in exon 1 of 12; may trigger nonsense-mediated decay and potentially reduce functional SLC35D1 dosage; not experimentally validated
SLC35D1	TCGA-AA-3516-01	Female	1	67515492	A	G	Missense Mutation	ENST00000235345	c.506T>C	p.Met169Thr	p.M169T	6/12	novel	deleterious	possibly damaging	Predicted to be deleterious by SIFT and possibly damaging by PolyPhen-2; the specific functional consequence has not been experimentally determined.
SLC35D1	TCGA-AA-A00J-01	Male	1	67507996	C	T	Missense Mutation	ENST00000235345	c.652G>A	p.Gly218Arg	p.G218R	8/12	COSV52430133	deleterious	probably damaging	Predicted to be deleterious by SIFT and probably damaging by PolyPhen-2; the specific functional consequence has not been experimentally determined.
SLC35D2	TCGA-AG-A002-01	Male	9	99106263	C	T	Missense Mutation	ENST00000253270	c.607G>A	p.Gly203Arg	p.G203R	8/12	rs770468251	deleterious	possibly damaging	Predicted to be deleterious by SIFT and possibly damaging by PolyPhen-2; the specific functional consequence has not been experimentally determined.
SLC35D2	TCGA-AG-3901-01	Female	9	99084293	C	T	Missense Mutation	ENST00000253270	c.901G>A	p.Gly301Arg	p.G301R	11/12	COSV53565072	deleterious	probably damaging	Predicted to be deleterious by SIFT and probably damaging by PolyPhen-2; the specific functional consequence has not been experimentally determined.
SLC35D2	TCGA-AA-A00N-01	Male	9	99083606	C	A	Missense Mutation	ENST00000253270	c.932G>T	p.Arg311Ile	p.R311I	12/12	COSV53565026	tolerated	benign	Predicted to have a low likelihood of damaging protein function by SIFT and PolyPhen-2; functional effects cannot be excluded.
SLC35D3	TCGA-AA-3519-01	Male	6	137243677	G	T	Missense Mutation	ENST00000331858	c.111G>T	p.Gln37His	p.Q37H	1/2	COSV100090489	tolerated	benign	Predicted to have a low likelihood of damaging protein function by SIFT and PolyPhen-2; functional effects cannot be excluded.
SLC35D3	TCGA-AA-3977-01	Male	6	137245734	C	T	Missense Mutation	ENST00000331858	c.1151C>T	p.Ser384Leu	p.S384L	2/2	COSV100090378; rs75[illegible]	[illegible]ated (low confid[illegible]	possibly damaging	Predicted possibly damaging by PolyPhen-2 but tolerated (low confidence) by SIFT; functional consequence uncertain
SLC35D2	TCGA-AA-A010-01	Female	n/a	n/a	n/a	n/a	Amplification	n/a	n/a	n/a	n/a	n/a	n/a	n/a	n/a	Copy-number alteration; no change to the coding sequence

Somatic mutations and copy-number alterations of SLC35D1, SLC35D2 and SLC35D3 identified in the TCGA Colorectal Adenocarcinoma cohort (Firehose Legacy; study identifier coadread_tcga; n = 223 samples with available mutation data), retrieved from the cBioPortal for Cancer Genomics (https://www.cbioportal.org) on 27 July 2026.

Genomic coordinates refer to genome build GRCh37/hg19. HGVS nomenclature is given relative to the canonical Ensembl transcript. Known variant identifiers were obtained from dbSNP and COSMIC; "novel" indicates that no matching entry was found.

SIFT and PolyPhen-2 predictions were generated with the Ensembl Variant Effect Predictor and are computational in silico predictions that have not been experimentally validated.

Abbreviations: HGVS, Human Genome Variation Society; n/a, not applicable.
