# Supplementary material for: SLC35D1 Is Associated with Colorectal Cancer Progression, EMT-Related Phenotypes, and Immune Infiltration
Source: Int J Mol Sci. 2026 Sep 24;27(19):8517. doi: 10.3390/ijms27198517 (PMC13664988; doi:10.3390/ijms27198517)
Supplement: Supplementary file 1 [file ijms-27-08517-s001.zip › Table S2.pdf]

Supplementary Table S2. Sex-stratified frequencies of SLC35D family gene alterations in colorectal cancer (TCGA COADREAD, Firehose Legacy).

Gene	Male, n/N (%)	Female, n/N (%)	P value
SLC35D1	2/116 (1.72)	1/107 (0.93)	1.000
SLC35D2	2/116 (1.72)	2/107 (1.87)	1.000
SLC35D3	2/116 (1.72)	0/107 (0.00)	0.499
Any SLC35D family gene	5/116 (4.31)	3/107 (2.80)	0.723

Alterations comprise somatic mutations and copy-number alterations of SLC35D1, SLC35D2 and SLC35D3 identified in the TCGA Colorectal Adenocarcinoma cohort (Firehose Legacy; study identifier coadread_tcga), retrieved from the cBioPortal for Cancer Genomics (https://www.cbioportal.org) on 27 July 2026. Individual variants are listed in Supplementary Table S3.

Denominators (N) are the numbers of male (N = 116) and female (N = 107) patients with available mutation data, not the full cohort; sex was recorded for all 223 sequenced patients. Percentages denote the proportion of patients of the indicated sex carrying an alteration. Patients carrying alterations in more than one gene are counted once per gene and once in the combined row.

P values were calculated by Fisher's exact test (two-sided). No comparison reached statistical significance. Because fewer than ten patients carried an alteration in total, these comparisons are exploratory and underpowered, and no correction for multiple comparisons was applied.

Abbreviations: n, number of patients carrying an alteration; N, number of patients assessed.
