# Supplementary material for: SLC35D1 Is Associated with Colorectal Cancer Progression, EMT-Related Phenotypes, and Immune Infiltration
Source: Int J Mol Sci. 2026 Sep 24;27(19):8517. doi: 10.3390/ijms27198517 (PMC13664988; doi:10.3390/ijms27198517)
Supplement: Supplementary file 1 [file ijms-27-08517-s001.zip › Table S3.pdf]

Table S3. Results of GSEA analysis

Pathway ID	enrichmentScore	NES	p.adjust	qvalue
HALLMARK_ALLOGRAFT_REJECTION	-0.52	-2.11	2.50E-09	1.47E-09
HALLMARK_INTERFERON_GAMMA_RESPONSE	-0.52	-2.10	2.50E-09	1.47E-09
HALLMARK_INTERFERON_ALPHA_RESPONSE	-0.50	-1.91	1.92E-04	1.13E-04
HALLMARK_APICAL_SURFACE	-0.48	-1.61	1.89E-02	1.11E-02
HALLMARK_MYOGENESIS	-0.46	-1.86	3.81E-06	2.25E-06
HALLMARK_IL6_JAK_STAT3_SIGNALING	-0.44	-1.66	8.45E-03	4.98E-03
HALLMARK_EPITHELIAL_MESENCHYMAL_TRANSITION	-0.44	-1.80	2.27E-05	1.34E-05
HALLMARK_COMPLEMENT	-0.41	-1.69	2.42E-04	1.42E-04
HALLMARK_COAGULATION	-0.41	-1.62	2.55E-03	1.50E-03
HALLMARK_KRAS_SIGNALING_DN	-0.40	-1.63	7.71E-04	4.55E-04
HALLMARK_APICAL_JUNCTION	-0.39	-1.60	7.12E-04	4.20E-04
HALLMARK_HYPOXIA	-0.39	-1.58	1.67E-03	9.83E-04
HALLMARK_INFLAMMATORY_RESPONSE	-0.39	-1.57	1.87E-03	1.10E-03
HALLMARK_DNA_REPAIR	-0.37	-1.48	1.67E-02	9.85E-03
HALLMARK_OXIDATIVE_PHOSPHORYLATION	-0.36	-1.45	1.89E-02	1.11E-02
HALLMARK_ADIPOGENESIS	-0.34	-1.39	4.00E-02	2.36E-02
HALLMARK_ANDROGEN_RESPONSE	0.29	1.38	4.17E-02	2.46E-02
HALLMARK_PROTEIN_SECRETION	0.38	1.79	7.10E-04	4.19E-04
