# Supplementary material for: SLC35D1 Is Associated with Colorectal Cancer Progression, EMT-Related Phenotypes, and Immune Infiltration
Source: Int J Mol Sci. 2026 Sep 24;27(19):8517. doi: 10.3390/ijms27198517 (PMC13664988; doi:10.3390/ijms27198517)
Supplement: Supplementary file 1 [file ijms-27-08517-s001.zip › Table S4.pdf]

Table S4. The results of hub gene screening using CytoNCA

NO.	Gene name	Degree	Betweenness	Closeness
1	AHSG	22	12520.56	0.04
2	APOB	21	5167.13	0.04
3	CCK	16	10946.05	0.04
4	APOE	16	7374.70	0.04
5	APOA4	15	4092.45	0.04
6	APOC3	15	2063.87	0.04
7	SPRR1B	14	3289.89	0.04
8	CSF2	14	10590.35	0.04
9	S100A7	13	4677.76	0.04
10	SAA1	13	5029.93	0.04
11	FABP4	13	8155.51	0.04
12	OXT	12	4403.40	0.04
13	GATA4	12	7979.25	0.04
14	PLG	12	2886.80	0.04
15	KRT6A	11	1561.98	0.04
16	KRT16	11	2042.80	0.04
17	PSCA	11	4929.66	0.04
18	AMH	11	3773.74	0.04
19	NKG7	10	3907.27	0.04
20	LHX1	10	3249.43	0.04
21	NR5A1	10	1789.02	0.04
22	FSHR	10	4119.64	0.04
23	RBP4	10	2864.42	0.04
24	RETN	10	2608.92	0.04
25	PROK1	10	4346.02	0.04
26	ADCY8	10	8085.39	0.04
27	LYPD2	9	1869.55	0.04
28	KRT14	9	1171.85	0.04
29	MUC6	9	4924.38	0.04
30	CDKN2A	9	9344.08	0.04
31	IGFBP1	9	2129.45	0.04
32	GNAT3	9	5304.78	0.04
33	PRKCG	9	4051.58	0.04
34	TEX101	8	4723.54	0.04
35	KLK7	8	900.84	0.04

36	MSLN	8	4646.31	0.04
37	SLURP1	8	3265.27	0.04
38	CLPS	8	3777.78	0.04
39	GAST	8	3965.86	0.04
40	HSD17B1	8	1576.24	0.04
41	UGT2A3	8	4372.67	0.04
42	PAH	8	1015.67	0.04
43	CASR	8	4066.88	0.04
44	TM4SF4	8	2399.35	0.04
45	FTCD	8	1924.62	0.04
46	MAGEA1	8	3431.72	0.04
47	LY6K	7	225.60	0.04
48	KRT6B	7	73.22	0.04
49	MIOX	7	3986.20	0.04
50	LY6E	7	987.76	0.04
51	HOXC13	7	1781.19	0.04
52	IL4I1	7	2048.30	0.04
53	GNLY	7	826.67	0.04
54	IDO1	7	3412.41	0.04
55	SPRR2A	7	31.96	0.04
56	PGC	7	3513.52	0.04
57	CIDEA	7	1675.70	0.04
58	KRT7	7	2782.46	0.04
59	KRT4	7	2328.76	0.04
60	LAG3	7	2735.50	0.04
61	MLN	7	1269.16	0.04
62	CALB1	7	1997.51	0.04
63	APOC1	7	44.70	0.04
64	HBZ	7	1093.57	0.04
65	KRT13	6	759.61	0.04
66	SIM1	6	1076.67	0.04
67	MUC16	6	1261.28	0.04
68	KLK6	6	2241.50	0.04
69	UPK2	6	1672.19	0.04
70	HOXC9	6	1469.63	0.04
71	NTSR2	6	2431.07	0.04
72	CNDP1	6	2350.88	0.04

73	ZIC1	6	2858.79	0.04
74	CCL3	6	1273.26	0.04
75	SAA2	6	0.00	0.04
76	NR0B1	6	2478.76	0.04
77	INHA	6	722.14	0.04
78	SOHLH1	6	1008.99	0.04
79	MIF	6	3194.61	0.04
80	EEF1A2	6	3643.68	0.04
81	NME2	6	2598.15	0.04
82	CALML5	6	5049.27	0.04
83	PASD1	6	3747.70	0.04
84	LYNX1	5	23.22	0.04
85	LY6D	5	1038.62	0.04
86	KLK5	5	834.94	0.04
87	KISS1R	5	1955.34	0.04
88	HOXC11	5	100.50	0.04
89	MYH6	5	1626.86	0.04
90	FGF3	5	755.69	0.04
91	CTSW	5	3.80	0.04
92	GZMH	5	3.80	0.04
93	GJA3	5	1200.78	0.04
94	CRCT1	5	69.47	0.04
95	NKX6-2	5	1702.36	0.04
96	SFTPC	5	1725.56	0.04
97	MAPK12	5	2687.07	0.04
98	OPRM1	5	2796.98	0.04
99	HCN2	5	2192.34	0.04
100	FLG2	5	451.52	0.04
101	PLA2G2A	5	1203.85	0.04
102	WNT7A	5	939.09	0.04
103	KRT2	5	1542.72	0.04
104	BPIFB2	5	839.78	0.04
105	AVPR2	5	2005.52	0.04
106	REG3A	4	165.77	0.04
107	PIP5KL1	4	1201.33	0.04
108	LGALS7B	4	719.19	0.04
109	ISYNA1	4	1353.75	0.04

110	HOXC5	4	0.00	0.04
111	HOXC10	4	0.00	0.04
112	HCST	4	127.24	0.04
113	IRX4	4	1529.48	0.04
114	NAT8L	4	2237.92	0.04
115	FTH1	4	827.45	0.04
116	WNT10B	4	1161.38	0.04
117	SPHK1	4	1195.00	0.04
118	CYP1A1	4	1180.69	0.04
119	CST7	4	0.00	0.04
120	CRYBA2	4	969.51	0.04
121	SPRR2E	4	0.00	0.04
122	SPINK6	4	135.61	0.04
123	REG1B	4	261.65	0.04
124	REG1A	4	176.13	0.04
125	CPA1	4	943.74	0.04
126	EYA1	4	575.61	0.04
127	ELOVL3	4	448.12	0.04
128	PCSK1N	4	298.65	0.04
129	CGB8	4	863.93	0.04
130	TNFRSF18	4	12.99	0.04
131	KIR2DL1	4	93.79	0.04
132	CD7	4	1648.22	0.04
133	UNC13C	4	1890.00	0.04
134	S100G	4	1286.66	0.04
135	MUC7	4	632.00	0.04
136	LPPR3	4	1185.97	0.04
137	ZNF683	4	69.89	0.04
138	TNNI2	4	1157.13	0.04
139	LIPI	4	883.85	0.04
140	PLA2G2D	4	735.04	0.04
141	HBA1	4	256.10	0.04
142	MTTP	4	0.00	0.04
143	LHB	4	356.47	0.04
144	HES7	4	962.91	0.04
145	SERPINB2	4	1402.67	0.04
146	ALDOA	4	852.50	0.04

147	UGT2B11	4	505.65	0.04
148	RPE65	4	1709.13	0.04
149	GUCY2D	4	705.19	0.04
150	AIPL1	4	1190.04	0.04
151	DLK1	4	1251.83	0.04
152	HABP2	4	592.21	0.04
153	ENPP3	4	886.19	0.04
154	KCNQ2	4	2487.52	0.04
155	CT83	4	881.42	0.04
156	ACTL8	4	1182.58	0.04
157	REG3G	3	57.56	0.04
158	SFTPB	3	894.79	0.04
159	RNF17	3	3128.00	0.04
160	PRR18	3	1260.00	0.04
161	NTSR1	3	594.85	0.04
162	LY6G6D	3	23.22	0.04
163	RTN4RL2	3	632.00	0.04
164	SAGE1	3	146.32	0.04
165	SERPINB4	3	851.59	0.04
166	SPINK9	3	72.02	0.04
167	LGALS7	3	217.02	0.04
168	HBG2	3	0.00	0.04
169	HBQ1	3	0.00	0.04
170	RGS13	3	632.00	0.04
171	HCN1	3	347.34	0.04
172	GALNT9	3	0.00	0.04
173	PCBP1	3	215.22	0.04
174	HMX3	3	327.42	0.04
175	WNT10A	3	30.29	0.04
176	HAMP	3	1426.65	0.04
177	SNCG	3	1109.90	0.04
178	DRD4	3	782.94	0.04
179	UTF1	3	632.00	0.04
180	VGF	3	136.86	0.04
181	FEZF2	3	76.66	0.04
182	CSAG1	3	90.00	0.04
183	PITX3	3	129.77	0.04

184	MYOT	3	351.95	0.04
185	TMEM252	3	47.43	0.04
186	LIPF	3	661.15	0.04
187	CLDN8	3	276.16	0.04
188	CLDN18	3	975.81	0.04
189	CITED1	3	275.79	0.04
190	EREG	3	124.70	0.04
191	CGB7	3	231.93	0.04
192	CDH15	3	1562.99	0.04
193	CD70	3	0.00	0.04
194	KIR3DL1	3	41.06	0.04
195	CALY	3	1260.00	0.04
196	PANX2	3	704.67	0.04
197	TRPV6	3	26.66	0.04
198	DLX1	3	272.34	0.04
199	ODF3B	3	1262.00	0.04
200	ALOXE3	3	1199.63	0.04
201	FKBP2	3	615.93	0.04
202	TKTL1	3	676.76	0.04
203	AKR1C4	3	35.70	0.04
204	UBD	3	929.53	0.04
205	PRODH2	3	700.75	0.04
206	NUDT3	3	632.00	0.04
207	CLPSL1	3	632.00	0.04
208	SLC34A2	2	0.00	0.04
209	TMEM88B	2	0.00	0.04
210	TMEM145	2	0.00	0.04
211	POLR2I	2	180.78	0.04
212	PAGE4	2	632.00	0.04
213	ODF3	2	994.79	0.04
214	NKX6-3	2	632.00	0.04
215	TMPRSS15	2	94.66	0.04
216	TACSTD2	2	250.89	0.04
217	KRTAP3-1	2	177.72	0.04
218	KRT3	2	55.39	0.04
219	NTF4	2	398.90	0.04
220	MCEMP1	2	501.72	0.04

221	HOXC12	2	0.00	0.04
222	HCAR2	2	632.00	0.04
223	RGS21	2	0.00	0.04
224	TMPRSS12	2	2.00	0.00
225	L1CAM	2	639.29	0.04
226	SFTA2	2	632.00	0.04
227	DNER	2	65.62	0.04
228	MAGEA12	2	0.00	0.04
229	CRABP2	2	632.00	0.04
230	COMP	2	2.00	0.00
231	TUBB4A	2	632.00	0.04
232	UPK1B	2	0.00	0.04
233	CLCA4	2	632.00	0.04
234	PLIN4	2	0.00	0.04
235	CDH7	2	632.00	0.04
236	CCL26	2	87.97	0.04
237	CCL25	2	21.11	0.04
238	TAS2R46	2	153.83	0.04
239	RAMP1	2	750.32	0.04
240	LIN7A	2	632.00	0.04
241	CASKIN1	2	1260.00	0.04
242	TMEM151A	2	632.00	0.04
243	C2CD4C	2	504.18	0.04
244	IL23R	2	642.75	0.04
245	C1orf141	2	84.64	0.04
246	C14orf105	2	632.00	0.04
247	CLDN6	2	372.52	0.04
248	PAPOLB	2	1884.00	0.04
249	SLC6A15	2	632.00	0.04
250	BOLL	2	1260.00	0.04
251	VMO1	2	208.94	0.04
252	TMEM160	2	0.00	0.04
253	VAMP5	1	0.00	0.04
254	TUBA3C	1	0.00	0.04
255	TRPM3	1	0.00	0.04
256	USP26	1	0.00	0.04
257	SYT12	1	0.00	0.04

258	TUSC5	1	0.00	0.04
259	TMEM229A	1	0.00	0.03
260	SMTNL1	1	0.00	0.04
261	RTBDN	1	0.00	0.04
262	SBK2	1	0.00	0.04
263	SLC14A1	1	0.00	0.04
264	PRSS55	1	0.00	0.04
265	TGM6	1	0.00	0.04
266	PIWIL3	1	0.00	0.04
267	PNMT	1	0.00	0.04
268	VGLL1	1	0.00	0.04
269	SSTR5	1	0.00	0.04
270	OPN5	1	0.00	0.00
271	TYMP	1	0.00	0.04
272	NKX2-8	1	0.00	0.04
273	VPREB1	1	0.00	0.04
274	MSMB	1	0.00	0.04
275	NDUFS7	1	0.00	0.00
276	MRPL2	1	0.00	0.00
277	PAPL	1	0.00	0.04
278	MAPK8IP2	1	0.00	0.04
279	MAFA	1	0.00	0.04
280	LY6G6F-LY6G6D	1	0.00	0.04
281	LIPJ	1	0.00	0.04
282	TAX1BP3	1	0.00	0.04
283	KLHDC7B	1	0.00	0.04
284	KLC3	1	0.00	0.04
285	LIN28B	1	0.00	0.04
286	NACA2	1	0.00	0.04
287	HTR4	1	0.00	0.04
288	HSPB2	1	0.00	0.04
289	TRIM7	1	0.00	0.04
290	HDAC10	1	0.00	0.04
291	SLC5A8	1	0.00	0.04
292	GPR25	1	0.00	0.04
293	OR7D4	1	0.00	0.04

294	LGALS2	1	0.00	0.00
295	GALR3	1	0.00	0.00
296	GABRA4	1	0.00	0.04
297	SRXN1	1	0.00	0.04
298	FSTL3	1	0.00	0.04
299	FOXI3	1	0.00	0.04
300	FAM26F	1	0.00	0.04
301	FAM217A	1	0.00	0.00
302	FAM132B	1	0.00	0.04
303	ENPP7	1	0.00	0.04
304	NMRK2	1	0.00	0.04
305	PYDC1	1	0.00	0.04
306	DPCR1	1	0.00	0.04
307	DNMT3L	1	0.00	0.04
308	CTXN1	1	0.00	0.04
309	MAGEB2	1	0.00	0.04
310	PRTN3	1	0.00	0.04
311	RARRES3	1	0.00	0.04
312	CPA2	1	0.00	0.04
313	SLC26A2	1	0.00	0.00
314	SCX	1	0.00	0.00
315	TMIGD1	1	0.00	0.04
316	CGB5	1	0.00	0.04
317	GATA5	1	0.00	0.04
318	MIA	1	0.00	0.04
319	TEKT2	1	0.00	0.00
320	CCDC39	1	0.00	0.00
321	CALHM3	1	0.00	0.04
322	CACNG6	1	0.00	0.04
323	CA6	1	0.00	0.04
324	C6orf15	1	0.00	0.04
325	NBEAL1	1	0.00	0.00
326	C1QL1	1	0.00	0.00
327	FSIP2	1	0.00	0.00
328	C19orf26	1	0.00	0.00
329	PRSS21	1	0.00	0.04
330	TXNDC5	1	0.00	0.00

331	C12orf10	1	0.00	0.00
332	ENDOG	1	0.00	0.00
333	BIRC7	1	0.00	0.00
334	ATP6V0A4	1	0.00	0.04
335	ANXA8	1	0.00	0.04
336	ANKRD30A	1	0.00	0.04
337	ANKRD33	1	0.00	0.04
338	ANXA8L1	1	0.00	0.04
