# Supplementary material for: SLC35D1 Is Associated with Colorectal Cancer Progression, EMT-Related Phenotypes, and Immune Infiltration
Source: Int J Mol Sci. 2026 Sep 24;27(19):8517. doi: 10.3390/ijms27198517 (PMC13664988; doi:10.3390/ijms27198517)
Supplement: Supplementary file 1 [file ijms-27-08517-s001.zip › Table S5.pdf]

Table S5. Supplementary Table for Key Reagents, Antibodies, and Kits Used

**Category**	**Reagent / Item**	**Catalog No.**	**Manufacturer / Vendor**	**Additional Info**
Cell line	DLD-1 (human CRC cell line)	CCL-221	ATCC (Rockville, MD, USA)	RRID: CVCL_0248
Cell line	HCT 116 (human CRC cell line)	CCL-247	ATCC (Rockville, MD, USA)	RRID: CVCL_0291
Cell culture	RPMI 1640 medium			
Cell culture	Fetal bovine serum (FBS)		SAFC Biosciences (Lenexa, KS, USA)	10% (v/v)
Cell culture	Penicillin/Streptomycin		Life Technologies	100 IU/mL penicillin, 100 μg/mL streptomycin
Constructs & transfection	SLC35D1 shRNA (clone TRCN0000233169)	TRCN0000233169	National RNAi Core Facility, Academia Sinica (Taiwan)	Targets human SLC35D1, NM_015139, (F)TGGCAGTTCTCTGGGTGGGAAA (R)GCTGAACAGTCCCGTGATTTGG
Constructs & transfection	Non-target (control) shRNA	SHC002	Sigma-Aldrich	5´-CAACAAGATGAAGAGCACCAA-3´
Constructs & transfection	pCMV6-Entry-SLC35D1 (overexpression plasmid)	RC203263	OriGene Technologies (Rockville, MD, USA)	
Constructs & transfection	Neon Electroporation System	Model MPK5000	Invitrogen (Life Technologies)	Used for transfection
Constructs & transfection	Puromycin (selection reagent)	ant-pr-1	InvivoGen	2-week selection of stable clones
SRB cell viability assay	Sulforhodamine B (SRB) reagent	S1402	Sigma-Aldrich	
Migration / invasion assay	BD Falcon™ cell culture inserts		BD Biosciences (Franklin Lakes, NJ, USA)	Migration assay
Migration / invasion assay	BD BioCoat™ Matrigel® Invasion Chambers		BD Biosciences (Franklin Lakes, NJ, USA)	Invasion assay
Migration / invasion assay	BD Matrigel Matrix		BD Biosciences (Franklin Lakes, NJ, USA)	Pre-coating of invasion chambers
Migration / invasion assay	Crystal violet stain	C0775	Sigma-Aldrich	0.1% (w/v)
Colony formation assay	Formaldehyde (fixative)	H121-08	Macron Fine Chemicals	0.4% (v/v)
Colony formation assay	Crystal violet stain	C0775	Sigma-Aldrich	0.1% (w/v)
Sphere formation assay	Basic FGF (bFGF)	100-18B-100UG	PeproTech®	10 ng/mL
Sphere formation assay	EGF	cyt-332	Prospec	20 ng/mL
Sphere formation assay	B27 supplement serum free	17504044	Thermo Fisher Scientific	1×

qRT-PCR	TRIZOL reagent (RNA extraction)		Invitrogen Life Technologies (Carlsbad, CA, USA)	
qRT-PCR	cDNA Synthesis Kit		Invitrogen Life Technologies (Carlsbad, CA, USA)	
qRT-PCR	SYBR Green Master Mix		Applied Biosystems	
Western blot	Anti-GAPDH primary antibody	IR3-8	iReal Biotechnology	
Western blot	Anti-SLC35D1 primary antibody	GTX85390	GeneTex	
Western blot	Anti-Fibronectin primary antibody	IR48-147	iReal Biotechnology	
Western blot	Anti-N-cadherin primary antibody	IR46-143	iReal Biotechnology	
Western blot	Anti-E-cadherin primary antibody	IR55-180	iReal Biotechnology	
Western blot	Anti-Vimentin primary antibody	IR45-137	iReal Biotechnology	
Western blot	Secondary antibody (HRP-conjugated)	IgG: 111-035-003	Jackson ImmunoResearch	
Western blot	PVDF membrane		GE Healthcare (Piscataway, NJ, USA)	
Western blot	ECL detection reagent		GE Healthcare (Piscataway, NJ, USA)	
Immunofluorescence	Anti-E-cadherin antibody (IF)	IR55-180	iReal Biotechnology	
Immunofluorescence	Fluorescent secondary antibody	02-15-06	Kirkegaard & Perry Laboratories	
Immunofluorescence	DAPI	910-3008	Chemometec	
Equipment	ABI 7500 FAST Real-Time PCR System		Applied Biosystems	
Equipment	VersaDoc 5000 imaging system		Bio-Rad Laboratories (Hercules, CA, USA)	
